# Development, Validation, and Field-Testing of an Instrument for Clinical Assessment of HIV-Associated Neuropathy and Neuropathic Pain in Resource-Restricted and Large Population Study Settings

**DOI:** 10.1371/journal.pone.0164994

**Published:** 2016-10-20

**Authors:** Yohannes W. Woldeamanuel, Peter R. Kamerman, Demetri G. A. Veliotes, Tudor J. Phillips, David Asboe, Marta Boffito, Andrew S. C. Rice

**Affiliations:** 1 Pain Research, Department of Surgery and Cancer, Faculty of Medicine, Imperial College London, London, United Kingdom; 2 Department of Neurology, Addis Abäba University School of Medicine, College of Health Sciences, Addis Abäba, Ethiopia; 3 Advanced Clinical Consultation & Research Center, Addis Abäba, Ethiopia; 4 Brain Function Research Group, School of Physiology, Faculty of Health Sciences, University of the Witwatersrand, Johannesburg, South Africa; 5 Division of Neurology, School of Clinical Medicine, Faculty of Health Sciences, University of the Witwatersrand, Johannesburg, South Africa; 6 The Pain Relief Unit, The Churchill Hospital, Oxford, United Kingdom; 7 HIV Medicine and Sexual Health, Chelsea and Westminster Hospital, London, United Kingdom; University of Würzburg, GERMANY

## Abstract

HIV-associated sensory peripheral neuropathy (HIV-SN) afflicts approximately 50% of patients on antiretroviral therapy, and is associated with significant neuropathic pain. Simple accurate diagnostic instruments are required for clinical research and daily practice in both high- and low-resource setting. A 4-item clinical tool (CHANT: Clinical HIV-associated Neuropathy Tool) assessing symptoms (pain and numbness) and signs (ankle reflexes and vibration sense) was developed by selecting and combining the most accurate measurands from a deep phenotyping study of HIV positive people (Pain In Neuropathy Study–HIV-PINS). CHANT was alpha-tested *in silico* against the HIV-PINS dataset and then clinically validated and field-tested in HIV-positive cohorts in London, UK and Johannesburg, South Africa. The Utah Early Neuropathy Score (UENS) was used as the reference standard in both settings. In a second step, neuropathic pain in the presence of HIV-SN was assessed using the Douleur Neuropathique en 4 Questions (DN4)-interview and a body map. CHANT achieved high accuracy on alpha-testing with sensitivity and specificity of 82% and 90%, respectively. In 30 patients in London, CHANT diagnosed 43.3% (13/30) HIV-SN (66.7% with neuropathic pain); sensitivity = 100%, specificity = 85%, and likelihood ratio = 6.7 versus UENS, internal consistency = 0.88 (Cronbach alpha), average item-total correlation = 0.73 (Spearman’s Rho), and inter-tester concordance > 0.93 (Spearman’s Rho). In 50 patients in Johannesburg, CHANT diagnosed 66% (33/50) HIV-SN (78.8% neuropathic pain); sensitivity = 74.4%, specificity = 85.7%, and likelihood ratio = 5.29 versus UENS. A positive CHANT score markedly increased of pre- to post-test clinical certainty of HIV-SN from 43% to 83% in London, and from 66% to 92% in Johannesburg. In conclusion, a combination of four easily and quickly assessed clinical items can be used to accurately diagnose HIV-SN. DN4-interview used in the context of bilateral feet pain can be used to identify those with neuropathic pain.

## Introduction

HIV-associated sensory peripheral neuropathy (HIV-SN) is a length-dependent distal symmetrical polyneuropathy that occurs in individuals with treated and untreated HIV infection. Increasing age and greater height appear to be universal risk factors for the neuropathy[1], while modifiable risk factors include: advanced AIDS and lower CD4 count in untreated infection, and exposure to stavudine-based ART[2–6], and perhaps protease inhibitors[2,7,8] in individuals receiving antiretroviral treatment (ART). However, even as less neurotoxic antiretroviral treatment regimens are introduced globally, the prevalence of the neuropathy is not expected to decrease appreciably[9–11].

The lack of a gold standard for accurately diagnosing HIV-SN makes proper prevalence assessment and disease management to be challenging^16^. Several groups have assessed the prevalence of HIV-SN, defined by the presence of both compatible neuropathic symptoms and signs. A recent study in a resource-limited setting in India found 40% prevalence of HIV-SN among untreated HIV-infected patients[12]. Studies from Africa report similar prevalence of HIV-SN, with 37% of treatment-naïve patients[13], and ~60% of treated patients having the neuropathy[13–15]. Moreover, the neuropathy commonly is painful. A study in South Africa reported over 70% of patients with HIV-SN had moderate-to-severe pain and paraesthesiae[14]. The pain from HIV-SN has a detrimental effect on quality of life[9,16–21] and adherence to ART[22].

Treating the pain, and treating it appropriately[23], requires identification of the pain as being neuropathic in origin[24,25]. To provide a definite diagnosis of neuropathic pain requires the use of specialized tests that assess large nerve fibers (quantitative sensory testing (QST)[26–28], nerve conduction studies (NCS)), and small nerve fibers (skin biopsy for analyzing intraepidermal nerve fiber density (IENFD)[29,30], QST). Assessments such as QST and IENFD require high levels of expertise, take a long time to perform, and are expensive, and therefore they are not routinely available, even in well-resourced settings. And, NCS, which is more widely available, cannot be used alone to exclude the presence of a neuropathy because HIV-SN predominantly affects small sensory fibers, and NCS assesses large fiber function only[29,31,32]. Thus, the diagnosis of painful HIV-SN typically relies solely on the outcome of a clinical history and/or examination. However, performing a comprehensive neurological examination requires time and specialist-level training to execute properly. Both these resources are typically unavailable in busy HIV clinics, which are staffed by primary care physicians, infectious diseases specialists, and nurses.

Neuropathy and neuropathic pain screening tools provide a possible solution. They provide an abbreviated, focused assessment that can be applied and interpreted quickly by non-experts after no or limited training[33]. Only two instruments, the Single-Question Neuropathy Screen (SQNS)[34], and the Brief Peripheral Neuropathy Screen (BPNS)[35], have been developed specifically for the assessment of HIV-SN. The SQNS only assesses symptoms and is thus simple and very quick to administer, but the trade-off is that it only yields a diagnostic certainty of possible neuropathic pain[34]. The BPNS is more robust than the SQNS and involves the bilateral assessment of clinical signs (ankle reflexes and vibration sense) and symptoms in the distal limbs[35]. While the focused clinical assessment in the BPNS means that it takes more time and some training to complete compared with the SQNS, the combination of clinical signs and symptoms yields a diagnostic certainty of probable neuropathic pain; the minimum level of certainty that indicates that the presence of the condition has been identified[24]. The utility of the BPNS was assessed by comparing the QST and IENFD results of HIV-infected individuals diagnosed as having or not having SN according to the BPNS. Unfortunately, although the sensitivity and specificity of individual items on the BPNS were assessed against the QST and IENFD data, validation of the BPNS scoring system, and the overall diagnostic sensitivity and specificity of the tool was not reported[35]. Moreover, in its original format, one of the items of this instrument, the ankle reflex, involved grading the reflex, which could be difficult for non-neurologists to assess accurately[35].

For the aforementioned reasons, there is an urgent need to develop simpler, economical, and robust clinical-based assessment tool requiring minimal training and expertise to deliver, and which has been validated against a gold standard diagnostic comprising of both clinical (i.e. symptoms and physical findings) and biological (i.e. epidermal innervation quantification) criteria rendering it to have a high diagnostic accuracy close to the ideal diagnostic methods. This clinical-based diagnostic tool will not only allow analgesic therapy to be appropriately directed, but will also be important in obtaining accurate diagnostic data for epidemiological and neuropathy risk factor studies, including large-scale genetic studies. Furthermore, such tool will be invaluable in ascertaining inclusion criteria for randomized controlled trials of suitable analgesics for neuropathic pain in both high- and low-resource settings, and assessing neurotoxicity profile of antiretroviral drugs. Developing, validating, and field-testing this tool was the objective underpinning our study described herein.

## Methods

### Ethics statement

Ethical clearance for the validation study conducted in London, UK: External Peer Review, application for UK National Health Service Research Ethical Committee (NHS REC) approval (REC Reference 13/NS/0090), NHS Research and Development (NHS R&D Number C&W13/058) approval, and National Institute for Health Research (NIHR Integrated Research Application System IRAS Ref: 127698. Study ID: 15040) Portfolio Adoption was sought and full approval was obtained. All participants provided written informed consent.

Ethical clearance for the field-test study in Johannesburg, South Africa was obtained from the Human Ethics Research Committee (Medical), University of the Witwatersrand (clearance number: M121018, issued: 12 July 2013). All participants provided written informed consent. Participants were compensated for their transport costs.

### Clinical Study Phases in Sequence (Fig 1A)

**Fig 1:**
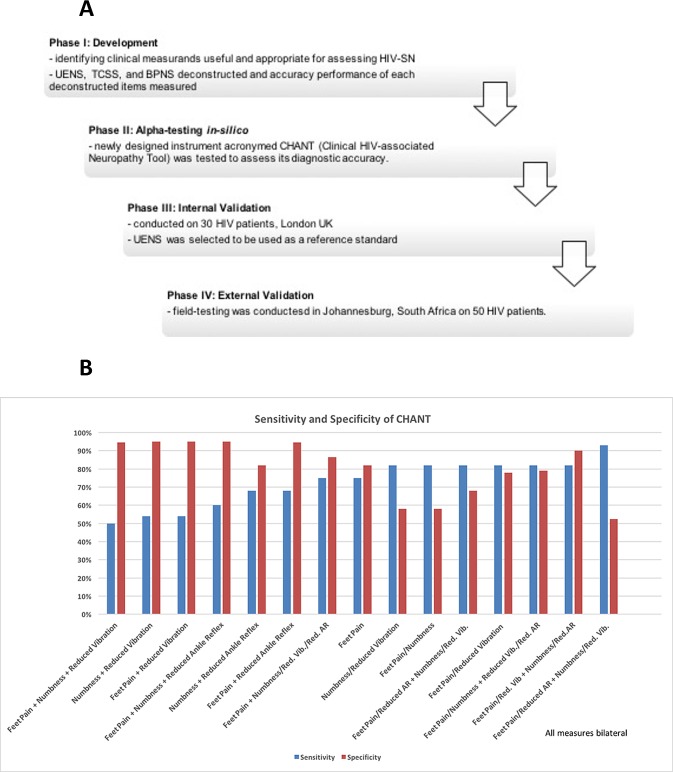
Development of the CHANT tool. (A) A summary flowchart describing important elements of our study phases in sequence. (B) Sensitivity and specificity of newly developed CHANT tool employing all possible cutoff combinations was conducted. The four-item CHANT was tested *in silico* within the stringent HIV-PINS diagnostic criteria so as to assess its accuracy performance for diagnosing neuropathy; this testing showed a high sensitivity and specificity of 82% and 90% respectively, while employing *‘bilateral feet pain/reduced great toe vibration’* AND *‘bilateral feet numbness/reduced ankle reflex’*. Hence, this cutoff was selected to diagnose HIV-SN when applying CHANT (Table 1). All measures were from bilateral feet.

The development of the screening tool involved two phases:

### Phase I: Development and Alpha-testing *in-silico*

- Development: Identification of clinical measurands from the Pain in Neuropathy Study–HIV (HIV-PINS)[20], a comprehensive phenotyping study of HIV-SN, that were judged as potentially useful and appropriate for routine clinical assessment of HIV-SN.
- Alpha-testing *in-silico*: The newly developed instrument, named the **C**linical **H**IV-**a**ssociated **N**europathy **T**ool (CHANT), was tested *in silico* within the HIV-PINS dataset to assess its diagnostic accuracy.

### Phase II: Validation

- Internal validation: Internal validation and field-testing of CHANT was conducted in 30 HIV-infected patients (outside the HIV-PINS study) at the Kobler Clinic, St. Stephen’s Centre, Chelsea and Westminster Hospital NHS Foundation Trust, London, UK.
- External validation and field-testing: External validation and field-testing was conducted in 50 HIV-positive patients attending an outpatient clinic at Chris Hani Baragwanath Academic Hospital, Johannesburg, South Africa.

### Phase I: Development and alpha-testing of Clinical HIV-associated Neuropathy Tool (CHANT)

#### Methods of Phase I

The development phase followed the triage of: i) assessing for the presence of peripheral neuropathy, and ii) assessing whether pain co-occurring with a neuropathy was primarily neuropathic in origin (NeP), iii) assessing severity and characteristics of present feet pain, and iv) assessing impact of present feet pain (Table 1). To assess the first triage stage, we looked to identify elements from the HIV-PINS dataset (Pain in Neuropathy Study–HIV[20]) that offer the best accuracy for identifying HIV-SN. The HIV-PINS dataset includes comprehensive sensory and psychological phenotyping data of 66 HIV-positive patients attending the Kobler Clinic, St. Stephen’s Centre, Chelsea and Westminster Hospital, London. The cohort consisted of patients with no evidence of neuropathy and patients with neuropathy (with and without neuropathic pain, NeP). The prevalence of neuropathy and neuropathic pain among the HIV-PINS cohort were compatible with the epidemiological data^16^. The HIV-PINS case definition for HIV-SN was made of 2 or more out of the following 3 items: clinical signs of distal sensory neuropathy elicited using the structured neurological examination, 2 or more abnormal QST findings using the full 13 parameters of the DFNS protocol, and intraepidermal nerve fibre density of ≤7.63 fibers/mm on skin sample examination, meets the requirements for identifying definite neuropathic pain proposed by Treede and colleagues[24] and those recommended by Devigili and colleagues[36]. The HIV-PINS phenotyping data included demographic data, clinical history, information about HIV and antiretroviral status, multiple questionnaires relating to neuropathy, pain, pain co-morbidities, and neurologic phenotype assessed using a structured neurological examination, UENS, BPNS, TCSS, the full German Neuropathic Pain Network Quantitative Sensory Testing (DFNS) QST protocol[27], and IENFD.

**Table 1:** Description of the characters of the four selected measurand components of the neuropathy tool.

	**AUC on ROC analysis (Area Under Curve on Receiver Operator Characteristic analysis)**	**Sensitivity and Specificity on cut-off Crossover trade-off between maximally high sensitivity and specificity**	**Partial Eta-squared value The variability in the dependent variable (presence or absence of neuropathy) can be explained or accounted for by the independent variable (e.g. feet pain); variables with higher values of partial Eta-squared carry more weight in defining neuropathy.**	**Geometric mean cut-off valueComputed as geometric mean of the four different methods of selecting cut-off i.e. specificity/sensitivity crossover positive and negative predictive value crossover positive and negative likelihood ratio crossover minimal cost of misdiagnosis**	**Cost of misdiagnosisApplying a false positive to false negative ratio of 0.50**
**Feet Pain**	0.79	69%	0.32 (Presence or Absence of painful feet)	≥ 5.5 [interpreted as mild to moderate on pain scale of 0–10]	Cost = 0.318
**Feet Numbness**	0.80	72%	0.29 (Presence or Absence of numb feet)	≥ 2.55 [interpreted as mild on 0–10 scale]	Cost = 0.364
**Vibration**	0.78	77.2%	0.264	≤ 13.2 seconds	Cost = 0.306
**Ankle reflex**	0.75	71.0%	0.289 (Bilaterally Reduced/Absent Ankle Reflex)	> 5 [interpreted to a value between ‘normal’ and ‘hypoactive’ ankle reflex]	Cost = 0.318

Components of HIV-PINS that were judged as potentially useful and neurologically appropriate for routine clinical assessment of HIV-associated sensory neuropathy (HIV-SN) and NeP were extracted. Similarly, potentially essential and useful components appropriate for routine clinical assessment of HIV-SN and NeP in resource-restricted and large population study settings were identified. This process was systematically performed based on a tactic of deconstruction and testing accuracy performance of individual components of different tools employed within HIV-PINS, so as to identify the most accurate measurands. Composite statistical analysis included detailed ROC performance for measuring diagnostic accuracy, partial Eta squared (*η*2) for computing effect size in ANCOVA (Analysis of Covariance) analysis so to indicate the accountability weight carried by each of the measurands, specificity and sensitivity calculations to derive the true positive and negative rates, cutoff selection using different decision level thresholds, and all families of *t*-tests where applicable.

Four cutoff decision thresholds were employed as crossover points between the curves of specificity and sensitivity, positive and negative predictive values, positive and negative likelihood ratios, and minimal cost of misdiagnosis; the geometric value of these four methods was used so as to develop a robust mean cutoff with central tendency. With regards to sensitivity and specificity, the point where the sensitivity and specificity curves cross was selected as the optimum decision level (i.e., where the maximum number of cases are correctly diagnosed as positive or negative). Similarly, the optimum decision level for likelihood ratios was chosen as the point where the curves of the positive and negative likelihood ratios crossover. The crossover point between higher values for positive likelihood ratio and lower values for negative likelihood ratio offer a better diagnostic accuracy (i.e., where the maximum number of cases being associated or not associated with having HIV-SN). Likewise, the optimum decision level for predictive values was chosen as the point of crossover between positive (where higher values mean better accuracy) and negative (where lower values mean better accuracy) predictive values. Misdiagnosis “cost” was taken into account as the fourth method of cutoff selection. Considering the higher cost impact of missing a false positive (FP) HIV-SN case, we applied relative misdiagnosis costs of 1 for true positives (TP), 1 for true negatives (TN), 1 for false positives (FN), and 2 for false negatives (FN), producing an FP:FN ratio of 0.5. Values >0.5 indicate increasing cost. Curve plots of TP, TN, FP and FN depending on chosen cutoff value was prepared to provide a general visual picture of how cutoff selection influences chances of having false tests of positives or negatives. An ideal test would have a cutoff that discriminates TP from TN without the presence of FP and FN, i.e. at the peak of each TN evolution curve. Statisitical softwares used were SigmaPlot 10.0, SPSS statistics 21, Microsoft Excel + Analyse-It 3.76, and GraphPad Prism 6.

Finally, the best components identified from the HIV-PINS dataset were refined, the question wordings improved, and the scorings simplified to generate binary outcomes. All outcomes of CHANT were designed to result in dichotomous scores of either present (*‘0’*) or absent (*‘1’*) and tallied for both *‘right’* and *‘left’* feet (reflecting the symmetrical presentation of HIV-SN) to simplify assessment and scoring. These components were combined to construct an optimal diagnostic instrument, designed to require minimal training and expertise to deliver.

In preparation of CHANT, HIV-PINS data from three previously validated tools for diagnosing peripheral neuropathy, in the context of diseases other than HIV infection, were deconstructed and explored so as to identify items and cutoff values which yielded high accuracy: Brief Peripheral Neuropathy Screen (BPNS)[35], Utah Early Neuropathy Score (UENS)[37] and Toronto Clinical Scoring System (TCSS)[38]. Of these three tools, the BPNS was specifically developed for use in the setting of HIV-SN while UENS and TCSS were developed for diabetic peripheral neuropathy^35-37^. Utility assessment of these three instruments was made in order to select the most accurate among the three for diagnosis of HIV-SN and to compare outcomes to the newly developed tool (see Phase II). The cutoff scores for these three instruments were prepared by computing geometric means for crossover values of sensitivity and specificity, positive and negative predictive values, positive and negative likelihood ratios, and cost of misdiagnosis[39]; the reference standard was the HIV-PINS case definition. The case definitions for HIV-SN used in these three tools is summarized in S1 Panel.

The second triage question was *“pain co-occurring with a neuropathy was primarily neuropathic in origin*”. To address this question, we used the interview component of Douleur Neuropathique en 4 Questions (DN4)[40]. DN4-Interview has been selected given its 78% sensitivity and 81.2% specificity rate of accurately diagnosing NeP when a score of ≥3 is employed[40], and its validation in several languages[41,42]. Since the DN4 has not previously been used to diagnose NeP within the setting of HIV-SN, this study has hence assessed its validity. Study patients were given a body chart to mark for area of pain; this helped confirm pain of plausible neuroanatomical distribution i.e. in this case, distal symmetrical pain distribution compatible with distal symmetrical polyneuropathy.

To address the third triage question *“What are the severity and characteristics of the present pain*?*”* the following instruments were employed: the Numeric Rating Scale (NRS)[43] to assess feet pain severity, and the Neuropathic Pain Symptoms Inventory (NPSI)[44] to characterize the feet pain symptoms. To address question #4 *“What is the impact of pain*?*”*, the Brief Pain Inventory (BPI)[45] was used to assess for pain interference to activities of daily living, the Depression Anxiety Positive Outlook Scale (DAPOS)[46] for brief assessment of psychological comorbidity, and the Insomnia Severity Index (ISI)[47] to assess overall sleep quality.

### Phase II: Validation of the developed clinical tool (CHANT)

#### Methods of Phase II

A validation study of CHANT was conducted using the UENS as the reference standard. A convenience sample of 30 HIV-infected out-patients (outside the HIV-PINS cohort) at the Kobler Clinic, St. Stephen’s Centre, Chelsea and Westminster Hospital, London, UK during August and September, 2013 was recruited. Between 5–10 participants per measurement item is deemed adequate to validate internal consistency correlations of construct validity[47], hence, a pilot sample size of 30 patients was adequate to clinically validate the 4-item instrument CHANT. Inclusion criteria were: consenting HIV-positive patients, who were 18 years or older, and who adequately understood verbal and written English. Individuals with concurrent severe psychological or psychiatric disorders, and those with central nervous lesions were excluded. The study investigator (YWW) was masked to any previous diagnosis of neuropathy. YWW conducted test-retest reliability of CHANT using the same experiment tools under the same conditions, in the same location repeated within the same day within a short period of time. A second study investigator (ASCR) and physicians (HY, AJ, GJ) also administered CHANT to each participant to allow the assessment of inter-tester concordance. The two investigators were blinded to each other’s assessment.

The validation study was conducted following a triage in the manner explained within *‘Methods of Phase I’* part of this manuscript i.e. assessing peripheral neuropathy, and assessing whether the present feet pain in neuropathy is from NeP, assessing severity and characteristics of present feet pain, and assessing impact of present feet pain. Face validity, content validity ratio, inter-tester reliability, internal consistency, predictive values, likelihood ratio, and pre- to post-test clinical probability for HIV-SN were analyzed. Likelihood ratios were emphasized as they appropriately summarize CHANT as a diagnostic test by combining sensitivity and specificity values. Fagan’s nomogram[48,49] was applied as it provides a practical graphic and user-friendly illustrative representation where likelihood ratios can be utilized along with a patient’s pre-test clinical probability of HIV-SN to estimate the post-test probability of HIV-SN. Given its high accuracy as described within *Results* section of *Phase I*, the UENS was selected as the reference standard to validate CHANT against; precaution was taken to distribute the UENS cutoff score symmetrically over both lower limbs, and exclude cases with high unilateral scores.

Following the positive internal validation study in London (see Results section of Phase II), an external validation and field-testing was conducted in at Chris Hani Baragwanath Academic Hospital, Johannesburg, South Africa, using the UENS as the reference standard similar to the study in London. A convenience sample of 50 HIV-positive out-patients was recruited. Like the London internal validation, the CHANT, UENS and the DN4-I (with a body map) were used to assess for the presence of HIV-SN and neuropathic pain. To mimic a research setting, participants were administered CHANT and the DN4-I (with body map) by a clinical researcher (PRK) trained in the assessment of ankle reflexes and vibration sense. The tools were administered in English, and participants were asked whether they understood the questions they were asked and the description of the tests that were going to be performed. When participants did not understand a question an interpreter, fluent in local indigenous languages, clarified the question before the participant answered in English or their home language. Participants also were asked whether they would prefer to have the screening tools administered in English or their home language. On a separate occasion, participants returned to the hospital and underwent a neurological examination of the upper and lower limbs, which included all items assessed by the UENS. The assessment was performed by a registrar in neurology (DGAV), who was blind to the outcome of the assessment with CHANT. Data analysis followed that described for the London validation study.

Face validity of CHANT was assessed after the instrument was circulated among all users and neurology experts involved within this study. Content Validity Ratio (CVR)[50,51] of CHANT was assessed to measure the correspondence between the four measurand items and the symptom content of HIV-SN. This was conducted by collecting responses of the following questions from five subject matter expert (SME) panelists which included one general practitioner, two neurologists, one pain medicine specialist, and one neuroscientist. The questions were asked to indicate one response whether each of the CHANT items *is 'essential*,*' 'useful*, *but not essential*,*' or 'not necessary' to the performance of CHANT*. CVR was then computed as CVR = (n_e_—N/2)/(N/2)[50,51] where n_e_ = number of subject matter expert panelists indicating "essential", N = total number of panelists. This formula yields values that range from +1 to -1; positive values indicate that at least half the panelists rated the item as essential.

## Results of Phase I

**Fig 2:**
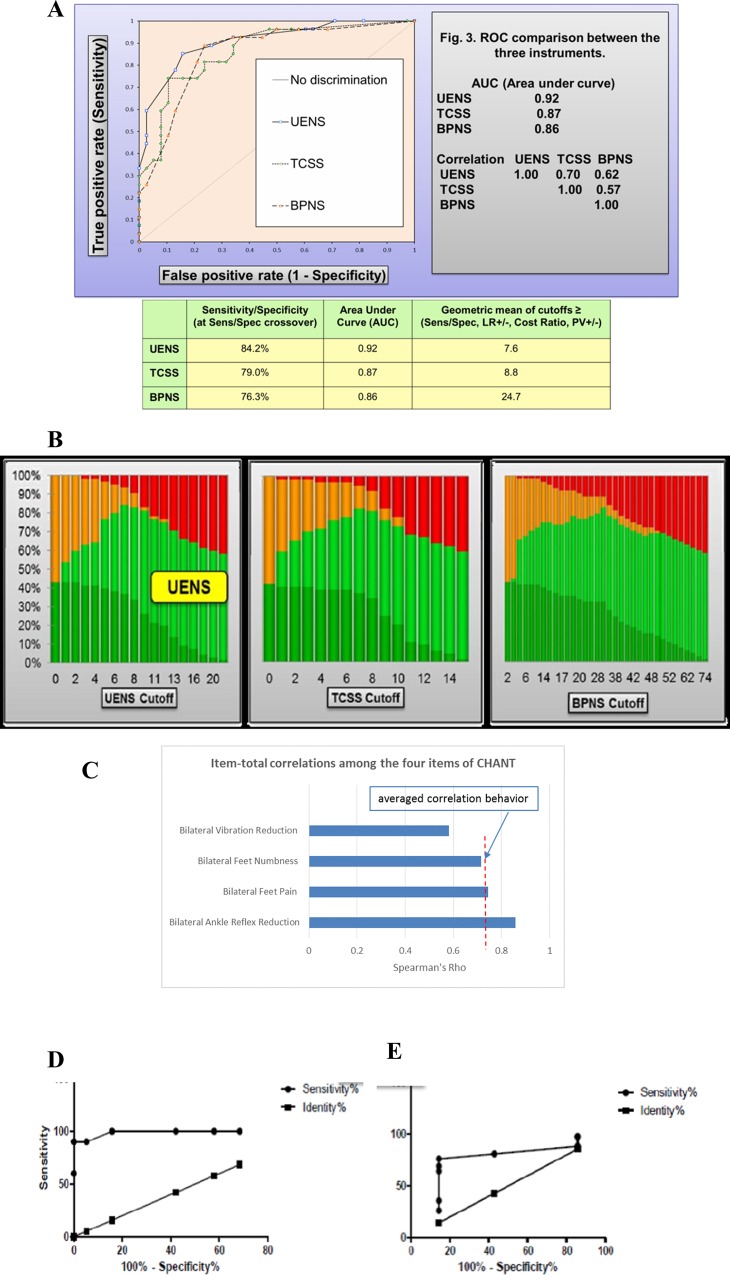
Alpha-testing of CHANT *in-silico*. (A) ROC comparison and optimum cutoff scores were analyzed among the three commonly employed neuropathy tools i.e. UENS, BPNS, TCSS—using the HIV-PINS diagnostic criteria as gold standard. Whilst all three instruments show utility for diagnosing HIV-SN, UENS has the highest accuracy, and hence selected as gold-standard for CHANT validation study. To enable bilateral assessment, the UENS cutoff of ‘8’ was distributed symmetrically between the right and left feet. The diagonal line depicts a performance where the specific instrument is unable to diagnose HIV-SN cases better than chance. (B) Evolution of counting TP (true positives), TN (true negative), FP (false positives) and FN (false negatives) was examined depending on chosen cutoff value of BPNS, TCSS, and UENS. This figure provides a general visual picture of how cutoff selection influences chances of having false tests of positives or negatives. An ideal test would have a cutoff that discriminates TP from TN without the presence of FP and FN, i.e. at the peak of each TN (light green curve). (C) Item-total correlations was analyzed among the four items of CHANT. Item-total correlations showed averaged behavior of 0.73 on Spearman’s Rho. Each item was consistent with the averaged behavior of all four items with very good discrimination performance, providing empirical evidence that all items represent the construct in CHANT, and merited to be included into the composite measurement. (D) ROC analysis of CHANT performance over UENS revealed high accuracy with area-under-curve (AUC) of 0.9895 in London validation study and 0.75 in Johannesburg field-testing study (E).

S1 Fig and Table 2 shows the diagnostic sensitivity and specificity, and S2 Fig shows the effect size of individual measurands from the HIV-PINS phenotype dataset, with the HIV-PINS case definition used as the reference standard. Our analysis identified two subjective questions of symptoms and two objective tests of clinical signs of neuropathy that yielded the best compromise between diagnostic accuracy and clinical utility. The two subjective items were *‘bilateral feet pain’* (sensitivity = 74%, and specificity = 81%) and *‘bilateral feet numbness’* (sensitivity = 77.8% and specificity = 63.2%) (S1 Fig, Table 2). The two objective items were *‘bilaterally reduced great toe vibration sense’* (sensitivity = 74% and specificity = 81%) and *‘bilaterally reduced ankle reflex’* (sensitivity = 85.7% and specificity = 68.4%) (S1 Fig, Table 2). These four items were used to construct the prototype four-item Clinical HIV-associated Neuropathy Tool (CHANT, complete description of the statistical characters of these four selected measurand components of CHANT are described in Table 2). The prototype was tested *in silico* within the HIV-PINS dataset, against the HIV-PINS reference standard, to assess its accuracy for diagnosing neuropathy. This testing showed a sensitivity and specificity of 82% and 90% respectively, while employing *‘presence of bilateral feet pain* or *reduced great toe vibration’* AND *‘bilateral feet numbness* or *reduced ankle reflex’* (Fig 1B). Hence, this cutoff was selected to diagnose HIV-SN when applying CHANT (Fig 1B, Table 2). A prototype tool (CHANT—Clinical HIV-associated Neuropathy Tool; Fig 2A, S2 Panel) was created by combining and refining the clinical phenotypes that achieved the best compromise between sensitivity and specificity.

**Table 2:** Rubric on scoring the CHANT tool. These combinations of values represent all possibilities of CHANT+ve neuropathy test results. The four-item CHANT was tested *in silico* within the stringent HIV-PINS diagnostic criteria so as to assess its accuracy performance for diagnosing neuropathy; this testing showed a high sensitivity and specificity of 82% and 90% respectively, while employing a minimum of *‘bilateral feet pain/reduced great toe vibration’* AND *‘bilateral feet numbness/reduced ankle reflex’* (Fig 4). Hence, this cutoff was selected to diagnose HIV-PN when applying CHANT.

Bilateral Feet Pain	Bilateral Feet Numbness	Bilateral Vibration at Great Toe	Bilateral Ankle Reflex	CHANT +ve i.e. ‘bilateral feet pain/reduced great toe vibration’ AND ‘bilateral feet numbness/reduced bilateral ankle reflex’ (all other scores remain CHANT–ve)
2	2	0	0	CHANT +ve
2	2	1	0	CHANT +ve
2	2	0	1	CHANT +ve
2	2	1	1	CHANT +ve
0	2	2	0	CHANT +ve
1	2	2	0	CHANT +ve
0	2	2	1	CHANT +ve
1	2	2	1	CHANT +ve
0	0	2	2	CHANT +ve
0	1	2	2	CHANT +ve
1	0	2	2	CHANT +ve
1	1	2	2	CHANT +ve
0	2	2	2	CHANT +ve
1	2	2	2	CHANT +ve
2	0	2	2	CHANT +ve
2	1	2	2	CHANT +ve
2	2	0	2	CHANT +ve
2	2	1	2	CHANT +ve
2	2	2	0	CHANT +ve
2	2	2	1	CHANT +ve
2	2	2	2	CHANT +ve

Among the three existing instruments (BPNS, UENS, and TCSS), the UENS had highest diagnostic accuracy (Fig 2A and 2B), and thus was selected as the reference standard for the validation and field-testing of CHANT (Phase II). Curve plots of counting true positives (TP), true negative (TN), false positives (FP) and false negatives (FN) depending on chosen cutoff value provided an overview of how cutoff selection influenced chances of having false tests of positives or negatives (Fig 2B); at the peak of each TN (light green curve on Fig 2B); the selected cutoffs from these three instruments of BPNS, UENS, and TCSS (Fig 2A) correlated with the peaks of each TN on the curves (Fig 2B).

## Results of Phase II

### London cohort

Complete data was collected from the recruited 30 patients. There were no missing items. Each assessment lasted around 45 minutes. Description of patient characteristics is available in S1 Table.

**Fig 3:**
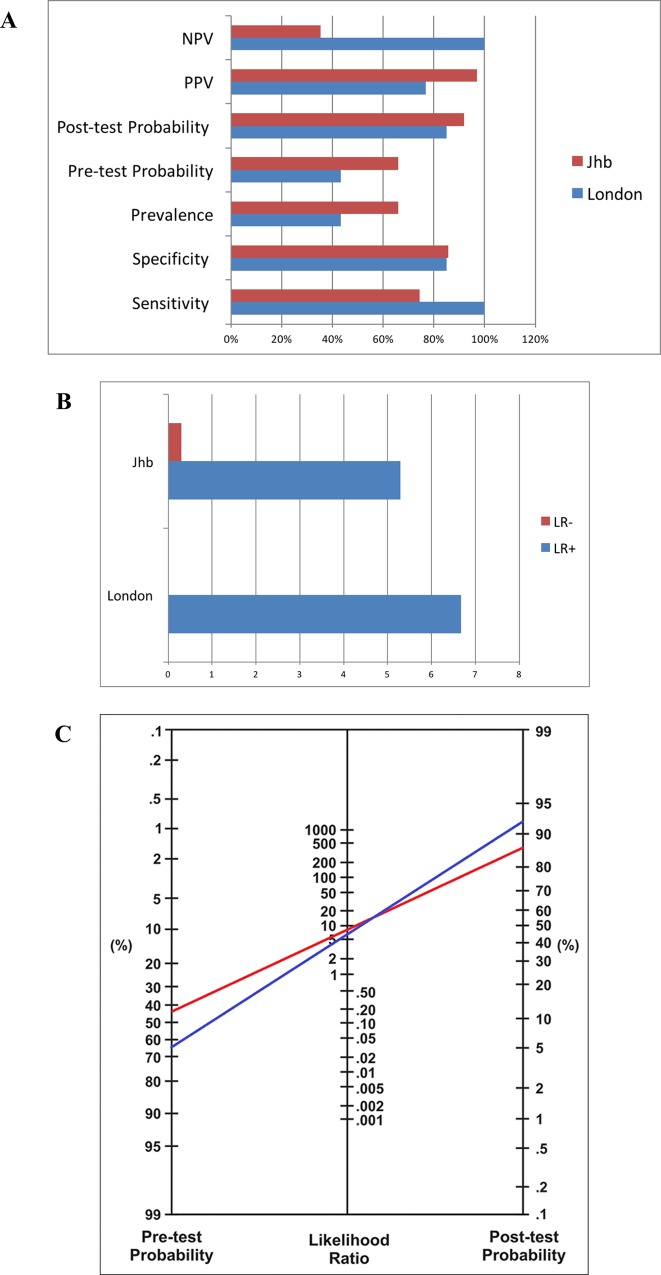
Validation of CHANT employing UENS as gold standard. In London CHANT validation study, no patient diagnosed using the UENS was missed by the CHANT; this resulted in sensitivity of 100%, specificity of 85%, PPV (positive predictive value) of 77%, NPV (negative predictive value) of 100% (A), and LR+ (positive likelihood ratio) of 6.67, and LR- (negative likelihood ratio) of 0 (B). In Johannesburg CHANT field-testing study, CHANT had sensitivity of 74.42%, specificity of 85.71, PPV of 96.97%, NPV of 35.3% (A), LR+ of 5.29, and LR- of 0.30 (B). Both the validation and field-testing revealed high accuracy. (C) Nomogram was made for interpreting the CHANT validation and field-testing diagnostic test results. In both circumstances of London (*red line*) and Johannesburg (*blue line*), a positive CHANT score has significantly increased clinical certainty of the patient having HIV-PN i.e. 43% to 85% in London (*red line*), and 66% to 92% in Johannesburg (*blue line*).

Out of the 30 patients, 13 (43%; 47% in males, 33% in females) had HIV-SN based on the CHANT tool; this was close to the neuropathy prevalence (42.42%; 28/66) reported among HIV-PINS patients at the same center, and using the stringent HIV-PINS reference standard[20]. Overall face validity of CHANT instrument among peer-reviewers, study investigators, untrained observers, and study patients was acceptable. No questions were identified as ambiguous or confusing. Subject matter experts rated each of the four items as being essential; content validity ratio (CVR) was 0.6 for ‘*feet pain’*, and 1 for the remaining 3 items, with a mean CVR of 0.9 across all four items. Spearman’s Rho (ρ) showed strong positive correlation for inter-tester reliability among all four components of CHANT (Fig 3C, Table 3). Internal consistency of CHANT was assessed using Cronbach’s α coefficient, and revealed a strong consistency of 0.88 (Fig 3C, Table 3) among its four different dichotomous components, indicating that the components of the multi-item CHANT scale measures the same concept. Item-total correlations showed averaged behavior of 0.73 on Spearman’s Rho (Fig 3C). Each item was consistent with the averaged behavior of all four items with very good discrimination, providing empirical evidence that all items represented the construct in CHANT, and merited inclusion (Fig 3C).

**Table 3:** London CHANT validation study: Reliability (inter-investigator), internal consistency, and item-total correlations. Spearman’s Rho showed strong positive correlation for inter-tester reliability among all four components of the CHANT. Internal consistency of CHANT was assessed using Cronbach’s α coefficient—and this revealed a strong consistency of 0.88 among its four different dichotomous components indicating that the multi-item CHANT scale measures the same concept, i.e. peripheral neuropathy.

CHANT	Reliability (Inter-tester) Spearman’s Rho (ρ)	Item-Total correlation Spearman’s Rho (ρ)
**Foot Pain (Right)**	1	Bilateral Feet Pain: 0.744
**Foot Pain (Left)**	1	Bilateral Feet Numbness: 0.717
**Foot Numbness (Right)**	1	Bilateral Vibration Reduction: 0.583
**Foot Numbness (Left)**	1	Bilateral Ankle Reflex Reduction: 0.858
**Vibration (Right)**	0.933	Averaged item-total correlation: 0.7255
**Vibration (Left)**	0.944	-
**Ankle Reflex (Right)**	0.968	-
**Ankle Reflex (Left)**	0.985	Internal Consistency Cronbach’s α: 0.8832

When comparing CHANT results to those from UENS, 10/30 patients had HIV-SN on UENS compared to 13/30 on CHANT. Receiver operating characteristics of CHANT using UENS as the reference standard revealed high accuracy with area-under-curve (AUC) of 0.99 (Fig 3D). One patient was found to have a high unilateral score on UENS, and was excluded. No patients diagnosed with HIV-SN using the UENS were missed by the CHANT (sensitivity = 100%), but 3 patients identified as not having HIV-SN when using UENS were classified as having SN when using CHANT (specificity of 85%). The positive predictive value (PPV) was 77%, negative predictive value (NPV) was 100%, and the likelihood ratio (LR) was 6.67 (Fig 3F and 3G). In order to interpret these diagnostic test results, a nomogram[49] was applied (*red line* in Fig 3H), according to which a positive CHANT result substantially increased the pre- to post-test clinical probability of the patient having HIV-SN from 43% to over 80% in a London tertiary-care setting.

**Fig 4:**
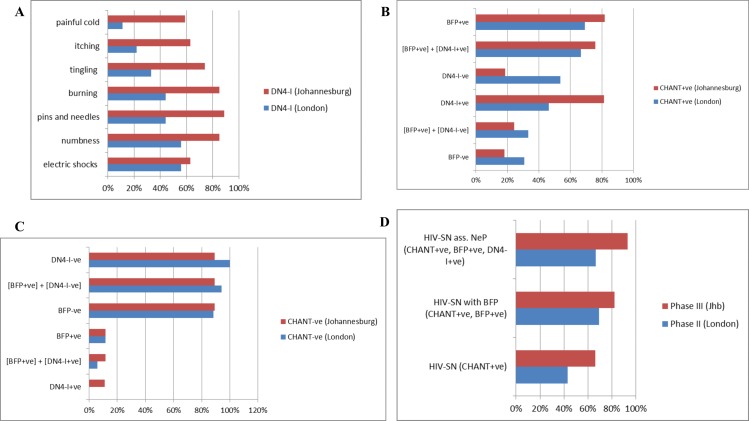
Features of HIV-SN in London and Johannesburg. (A) Frequency of symptoms from the DN4-Interview among the HIV-SN patients (CHANT+ve) with BFP (Bilateral Feet Pain) in London Internal Validation Study (*blue bars*) and in Johannesburg External Validation Study (*red bars*) was examined. Within the London cohort, nine of the 13 (69.23%) CHANT+ve neuropathy patients had bilateral feet pain on body map (BFP+ve), and 6 of these 9 (66.67%) had their DN4-interview (DN4-I) score ≥3/7 (median 5 (IQR: 3.5, 6.25)). Among these 9 patients, symptoms were described in the following decreasing frequency: *‘electric shocks’ (56%)*, *‘numbness’ (56%)*, *‘pins and needles’ (44%)*, *‘burning’ (44%)*, *‘tingling’ (33%)*, *‘itching’ (22%)*, *and ‘painful cold (11%)*. Additional descriptors used by patients to better explain their pain include: *‘tightness over feet and legs’*, *‘feels wearing tight-fitting shoes’*, and *‘cramps over feet and legs’*. Among the Johannesburg cohort (*red bars*), more than half of all the patients had all of the DN4-I symptom items, while ‘*pins and needles*’ was found to be most frequently present among 90% of the patients. (B) Distribution of symptoms of Bilateral Feet Pain (BFP), and DN4-I was evaluated among patients found to have HIV-SN as per the CHANT instrument in London Internal Validation Study (*blue bars*) and in Johannesburg External Validation Study (*red bars*). Among the London cohort (*blue bars*), close to 70% of these patients with HIV-SN had BFP and associated neuropathic pain ([BFP+ve] + [DN4-I+ve])–demonstrating the higher capacity of CHANT to identify BFP and associated neuropathic pain. Thirty % of those with HIV-SN (CHANT+ve) did not have BFP. Among the Johannesburg cohort (*red bars*), over 75% of these patients with HIV-SN had BFP and associated neuropathic pain ([BFP+ve] + [DN4-I+ve])–demonstrating the higher capacity of CHANT to identify BFP and associated neuropathic pain. Eighteen % of those with HIV-SN (CHANT+ve) did not have BFP. (C) Distribution of symptoms of Bilateral Feet Pain (BFP), and DN4-I was assessed among patients found *not* to have HIV-SN as per the CHANT instrument in London validation study (*blue bars*) and Johannesburg validation study (*red bars*). Over 80% of the London cohort patients *without* HIV-SN (*blue bars*) did not have BFP and had DN4-I-ve, while only 6% of these patients *without* HIV-SN were found to have associated bilateral feet neuropathic pain ([BFP+ve] + [DN4-I+ve]). Nearly 90% of the Johannesburg cohorts *without* HIV-SN (*red bars*) neither had BFP nor associated neuropathic pain ([BFP+ve] + [DN4-I+ve])–demonstrating the higher capacity of CHANT to identify BFP and associated neuropathic pain in this setting; only around 10% of those *without* HIV-SN (CHANT-ve) were found to have BFP and DN4-I+ve. (D) Comparison of HIV-SN, HIV-SN with bilateral feet pain (BFP), and HIV-SN associated bilateral feet neuropathic pain (NeP) was made between London and Johannesburg CHANT studies. In general, these results showed that there was moderately higher magnitude of both HIV-SN and HIV-SN associated neuropathic pain within the Johannesburg setting as compared to the setting in London; there was correlation evident between the three trends i.e. an increase in any of the three at one setting correlates to an increase in the other setting.

Nine of the 13 (69.2%) CHANT+ve neuropathy patients had bilateral feet pain on body map (BFP+ve,), and 6 of these 9 (66.7%) had DN4-interview (DN4-I) scores ≥3/7 (median 5, IQR: 3.5, 6.3) (Fig 4B, S2 Table). Among these 9 patients, distribution of DN4-I symptoms are shown on Fig 4A. Additional non-DN4 descriptors used by patients to better explain their pain include: *‘tightness over feet and legs’*, *‘feels wearing tight-fitting shoes’*, and *‘cramps over feet and legs’ (all at a similar rate of 3%)*. Over 80% of the patients *without* HIV-SN (CHANT-ve) did not have BFP and had DN4-I <3, while 6% of the patients *without* HIV-SN (CHANT-ve) had bilateral feet neuropathic pain ([BFP+ve] + [DN4-I+ve]) (Fig 4C, S2 Table). Permanent spontaneous feet pain (question #4 of NPSI) was present among 3 of the 9 (CHANT+ve, BFP) (33.33%), while 6/9 (66.67%) stated to have 6–10 episodes of feet pain attacks during the previous 24 hours (question #7) (S3 Table).

### Johannesburg cohort

Fifty patients were recruited. Patient characteristics are available on S1 Table. None of the participants spoke English as a first language. A language interpreter was required in 4% of assessments for the first CHANT item (‘*Do you have pain in your feet*?), 62% for the second CHANT item (‘*Do you have numbness in your feet*?), 12% for the third CHANT item (vibration test instructions), and 10% for the fourth CHANT item (ankle reflex test instructions) (S9 Fig). Overall, 42% of patients stated that they would prefer the instrument to be in their home language. When using the DN4-I, 30% of the patients required the assistance of an interpreter. Among the DN4-I items, those patients who required interpreter were 16% for the first item (‘whether the pain had *burning* characteristics’), 16% for the second item (‘whether the pain had *painfully cold* characteristics’), 26% for the third item (‘whether the pain had *electric shocks* characteristics), 81% for the fourth item (‘whether the pain had association with *tingling* symptoms in the same area), 52% for the fifth item (‘whether the pain had association with *pins and needles* symptoms in the same area), 77% for the sixth item (‘whether the pain had association with *numbness* symptoms in the same area), and 45% for the seventh item (‘whether the pain had association with *itchy* symptoms in the same area). Overall, 48% of the patients stated that they would prefer the instrument to be translated in their home language.

There was a 66% (33/50) prevalence of HIV-SN based on CHANT, out of which 26 (78.79%) had bilateral feet NeP on (DN4-I+ve, BFP on CHANT). Five of the eight males (62.5%) and 28/ 42 (66.7%) females were CHANT+ve. All 5/5 (100%) CHANT+ve males and 21/28 (75%) CHANT+ve females had NeP (DN4-I+ve, BFP). Clinical characteristics of participants with and without neuropathy on the CHANT tool are shown in S3–S8 Figs. When using the UENS, 43/50 (86%) had HIV-SN. Using the UENS as the reference standard, CHANT had a ROC AUC of 0.75 (Fig 3E), sensitivity of 74.42%, specificity of 85.71, PPV of 96.97%, NPV of 35.3%, and a LR of 5.29. A positive CHANT score markedly increased clinical certainty of the patient having HIV-SN from 66% (pre-test probability) to 92% (post-test probability) (Fig 3H).

Of those patients with HIV-SN (CHANT+ve), 82% (n/N) had BFP; 93% (n/N) of those with BFP had associated neuropathic pain (DN4-I scores ≥ 3/7; BFP+ve + DN4-I+ve; Fig 4E). Among the HIV-SN patients who had BFP, DN4-I symptoms were described in the following decreasing frequency: *‘pins and needles’ (89%)*, *‘burning’ (85%)*, *‘numbness’ (85%)*, *‘tingling’ (74%)*, *‘electric shocks’ (63%)*, *‘itching’ (63%) and ‘painful cold (59%)* (Fig 4A). Additional non-DN4 descriptors used by patients to better explain their pain included *‘cramps over feet and legs’*. Over 75% of the patients with HIV-SN (CHANT+ve) had bilateral feet neuropathic pain ([BFP+ve] + [DN4-I+ve]) (Fig 4B). Eighteen percent of those with HIV-SN (CHANT+ve) did not have BFP (Fig 4B). Nearly 90% of the patients *without* HIV-SN neither had BFP nor associated neuropathic pain ([BFP+ve] + [DN4-I+ve]). Only ~10% of those *without* HIV-SN (CHANT-ve) had BFP and DN4-I+ve (Fig 4C).

## Discussion

We undertook to develop and validate a screening tool for assessing HIV-SN that has high accuracy and clinical utility. CHANT was developed by combining four high-performing and easily assessed clinical measurands from the deep phenotyping HIV-PINS dataset. CHANT achieved high accuracy on alpha-testing with sensitivity and specificity of 82% and 90%, respectively. In 30 patients in London, CHANT diagnosed 43.3% (13/30) HIV-SN (66.7% with neuropathic pain); sensitivity = 100%, specificity = 85%, and likelihood ratio = 6.7 versus UENS, internal consistency = 0.88 (Cronbach alpha), average item-total correlation = 0.73 (Spearman’s Rho), high test-retest reliability = 0.85, and inter-tester concordance > 0.93 (Spearman’s Rho). In 50 patients in Johannesburg, CHANT diagnosed 66% (33/50) HIV-SN (78.8% neuropathic pain); sensitivity = 74.4%, specificity = 85.7%, and likelihood ratio = 5.29 versus UENS. A positive CHANT score markedly increased of pre- to post-test clinical certainty of HIV-SN from 43% to 83% in London, and from 66% to 92% in Johannesburg. DN4-interview used in the context of bilateral feet pain can be used to identify those with neuropathic pain.

The development of CHANT met the methodological quality criteria described by COSMIN (*Co*nsensus-based *S*tandards for the selection of health *M*easurement *In*struments)[52] for design requirements of clinical measurement properties (S3 Panel). However, developing screening tools for neuropathy and neuropathic pain is limited by the lack of gold standard diagnostic criteria. This means that the measures of validity and reliability we report for CHANT may over-estimate the true performance of the instrument. To minimize this error in Phase I of the tools development, we used a strict definition of neuropathy as our reference standard i.e. the HIV-PINS case definition for HIV-SN. In Phase II we did not have the required phenotype information to use this stringent case definition as the reference standard, and had to use a proxy measure, the UENS. The UENS was developed using a reference standard based on a case definition of similar stringency to that used for HIV-PINS[37]. The diagnostic properties of the UENS were confirmed in our study when we assessed it in our HIV-PINS cohort (Fig 3A). Nevertheless, the UENS is not 100% sensitive or specific, and thus we accept that the validity measures we report in Phase II are likely to be slightly inflated compared to if we had used a more stringent reference standard.

If the UENS performed well during Phase I, why do we not just use the UENS for assessing HIV-SN? Firstly, the UENS was not constructed to specifically address the symmetrical characteristics of HIV-SN, and therefore there is the potential for false positives for HIV-SN arising in unwary users as a result of high-scoring unilateral peripheral nerve lesion (e.g. radiculopathies). The CHANT was designed to overcome this limitation by keeping neuropathy symmetry central to its diagnostic criteria. Secondly, unlike CHANT, the UENS exclusively uses neurological examination items. The incorporation of signs and symptoms into the diagnostic definition of CHANT means that it is more closely aligned to the neuropathic pain grading system proposed by Treede and colleagues[24]. And lastly, while both tools include ankle reflexes and vibration sense, the UENS includes relative grading of both these items (normal, diminished, absent), whereas the CHANT records only presence and absence of the signs. This simplification may reduce the sensitivity of CHANT compared to the UENS, but it makes CHANT more readily usable by non-neurologists; one of our key aims. We acknowledge our limitation of not conducting test-retest reliability within the CHANT validation; we aim to include test-retest evaluations in the future.

The BPNS is the most frequently used screening tool for assessment of HIV-SN in the research setting, but the instrument’s overall sensitivity and specificity were never adequately described[35]. Nevertheless, there is strong convergence between the BPNS, whose development relied on clinical insight, and CHANT. Both assess the bilateral presence of two clinical signs (ankle reflexes and vibration sense) and symptoms (CHANT: pain and numbness; BPNS: pain, numbness, paresthesia). The most commonly used case definition of BPNS requires at least one bilateral sign and one bilateral symptom, while the case definition for CHANT can be made by based on the bilateral presence of two symptoms (pain and numbness) even if there are no signs present (Fig 2A).

Bilateral feet pain (BFP) was present among 69.23% of the neuropathy cases. While validating DN4-interview combining with body map pain distribution, it was found that 2/3^rd^ of these neuropathy patients with BFP were classified as having bilateral neuropathic foot pain (NeP), which compares to similar previous values found on applying more stringent and sophisticated criteria. HIV-SN is primarily a diffuse, non-focal PN; these features makes pain characteristics to be aberrant, and hence necessitates body map for identifying pain distribution along with DN4-Interview cutoff, or apply the DN4-Interview specific to *pain on bilateral feet*. The DN4-Interview was originally developed for focal NeP diagnosis, nonetheless our results showed its successful validation for HIV-SN NeP diagnosis. The three neuropathy cases with BFP but with no NeP need to be classified as Pain of Probably Neuropathic Origin (POPNO) until further confirmatory follow-up tests. It was interesting to note that foot pain was not a presenting feature among 4 of the 13 (30.77%) neuropathy cases. This supports the inclusion of *‘foot numbness’* and the two neurological examinations while constructing the CHANT tool. There was only one patient (3.33%) with no neuropathy where only BFP was present; he was referred for closer follow up as he may have presented with subclinical neuropathy. Three of the six patients (50%) with bilateral feet NeP had permanently persisting spontaneous pain, i.e. a classic feature of severity and chronicity in NeP. Two of the six (33.33%) NeP patients had dynamic mechanic allodynia over both feet.

Overall analysis of severity, characteristics, and impact of pain revealed a trend of progressive increment from no-neuropathy, to neuropathy, and to NeP. Application of body map was helpful in identifying pain specific to distal peripheral feet. NeP measurement instruments should employ descriptors mentioned by patients themselves e.g. *‘tightness over feet and legs’*, *‘as if wearing tight-fitting shoes’*, *‘cramps over feet and legs’*. NeP largely impacted lifestyles, particularly of sleep disturbance and pain interference with daily activities. The DAPOS scale was not discriminatory for psychological comorbidities between the no-neuropathy and neuropathy/NeP cases. This necessitates the design of a proper instrument that measures psychological comorbidities specific to patients with bilateral feet NeP, since concomitant presence of other somatic symptoms can influence outcomes. E-health resources can be used to develop smartphone application of the CHANT instrument for handy use among health professionals to help aid rapid diagnosis and triage.

We conducted validation and field-testing at two locations, London and Johannesburg. The selection of these cohorts allowed us to assess CHANT in a group of native English speakers and a group whose first language was not English. CHANT performed well in both cohorts, although some aspects of CHANT (especially the question on numbness) and the DN4-I (especially tingling and numbness) required the use of an interpreter in the Johannesburg cohort.

That CHANT had higher sensitivity and NPV in London validation compared to equivalent specificity and higher PPV in Johannesburg validation implies that CHANT can be utilized to rule *out* HIV-SN in the former, and rule *in* HIV-SN in the latter. This result fits to the characteristics of the two different settings in London and Johannesburg of our studies i.e. confirmatory tests are more available within the former setting compared to the later. Interpreting test results requires insight into the population for which the test is designed. Applying diagnostic or screening tests in low-prevalence settings can be misleading due to false positive results. One strategy to further improve the PPV of CHANT is to shift from screening everyone (universal screening) to screening selectively, e.g. testing only people with high risk (e.g. taller and older patients, patients on known neurotoxic drugs, or symptoms that suggest HIV-SN). A highlysensitive test such as CHANT within London setting will identify most cases of HIV-SN, so a CHANT-ve test reassures that the patient is least likely to have HIV-SN. Lower prevalence of HIV-SN in London means that a negative CHANT result is more accurate within that setting; higher HIV-SN prevalence in Johannesburg means that a positive CHANT result is more accurate within that setting. Application of tests to enhance the diagnostic likelihood leads to the notion of likelihood ratios. In both circumstances of London and Johannesburg, a positive CHANT score has markedly increased clinical certainty of the patient having HIV-SN i.e. 43% to 85% in London, and 66% to 92% in Johannesburg (Fig 3H). Negative CHANT test in London would definitely rule out HIV-SN, while a negative test in JHB will require re-evaluation follow-up if symptoms continue. A positive CHANT result gives higher clinical certainty of HIV-SN in Johannesburg. The HIV-SN patients with bilateral feet pain (BFP) in Johannesburg described more pain symptoms compared to those in London; this indicates that the clinical utility of this combination of CHANT, BFP, and DN4-I can be further enhanced as it helps to rule *out* HIV-SN associated bilateral feet neuropathic pain in Johannesburg (i.e. with a negative test result), while it can be used to rule *in* HIV-SN associated bilateral feet neuropathic pain in London (i.e. with a positive test result). In summary, all these results support the development of CHANT as an appropriate and accurate tool for diagnosing HIV-SN in large population and low resource clinical settings.

### Comparison of Results from London and Johannesburg

Comparison between the accuracy performance of CHANT within London and Johannesburg cohorts shows higher sensitivity in London and higher specificity in Johannesburg) (Fig 3F). Comparison of HIV-SN, HIV-SN with bilateral feet pain (BFP), and HIV-SN associated bilateral feet neuropathic pain (NeP) between London and Johannesburg CHANT studies revealed that there was moderately higher magnitude of both HIV-SN and HIV-SN associated neuropathic pain within the Johannesburg setting as compared to the setting in London; there was correlation evident between the three trends i.e. an increase in any of the three at one setting correlates to an increase in the other setting (Fig 4D). Comparison of frequency of DN4-I symptom items among the HIV-SN patients (CHANT+ve) with BFP (Bilateral Feet Pain) in London and Johannesburg Validation Studies showed that the patients in the Johannesburg cohort described more pain symptoms compared to those in London. Comparison of bilateral feet pain (BFP) and DN4-I scores between the patients in London and Johannesburg revealed slightly more number of patients with BFP and associated neuropathic pain (BFP+, DN4-I+) among the Johannesburg cohort; however the tool’s capacity to identify these patients was comparable in both settings (Fig 4D). The higher risk for pretest probability of having HIV-SN in African settings can make CHANT yield a high positive predictive value (PPV); this is because PPV results rely on pretest prevalence. In addition, differences in exposure to potentially neurotoxic antiretrovirals in Johannesburg and London may have influenced results.

## Conclusion

We found that combination of four clinical items is sufficient to accurately diagnose HIV-SN. CHANT provides a valid, reliable, practical, measurable, sensitive tool requiring minimal training and expertise to deliver for clinical research and daily practice. Assessment of inherent psychometric properties of CHANT demonstrated adequate performance. DN4-Interview along with body map is a valid tool to diagnose neuropathic pain among those with HIV-SN.

## Supporting Information

S1 Fig Sensitivity and specificity analysis of measurand items employed within the HIV-PINS (Pain in Neuropathy Study–HIV) dataset. The triumvirate HIV-PINS diagnostic criteria of clinical findings, QST (Quantitative Sensory Testing) abnormalities, and IENFD (Intraepidermal Nerve Fiber Density) results were used as gold-standard. Clinical items with high sensitivity and specificity i.e. feet numbness, feet pain, reduction in ankle reflex and great toe vibration were selected to construct CHANT (Clinical HIV-associated Neuropathy Tool). As shown herewith, these four clinical items performed accurately compared to most of the demanding neuropathy investigations. All items represent measures from bilateral feet.
(TIF)

S2 Fig Partial Eta-squared values of measurand items employed within the HIV-PINS (Pain in Neuropathy Study–HIV) dataset. The four clinical items with high sensitivity and specificity i.e. feet numbness, feet pain, reduction in ankle reflex and great toe vibration that were selected to construct CHANT (Clinical HIV-associated Neuropathy Tool) also carried higher accountability effect size compared to most of the demanding neuropathy investigations. All items represent measures from bilateral feet.
(TIF)

S3 Fig Age of participants stratified by diagnosis. In both cohorts of Johannesburg and London, there was no statistically significant increase in age (years) from no-neuropathy (CHANT-ve), to neuropathy (CHANT +ve), and to neuropathic pain (CHANT+ve, BFP+ve, DN4-I+ve) (Kruskal-Wallis followed by Dunn’s post-hoc analysis).
(TIF)

S4 Fig BMI (Body Mass Index) of participants stratified by diagnosis. In both cohorts of Johannesburg and London, there was no statistically significant difference in BMI among cases with no-neuropathy (CHANT-ve), neuropathy (CHANT +ve), and neuropathic pain (CHANT+ve, BFP+ve, DN4-I+ve) (Kruskal-Wallis followed by Dunn’s post-hoc analysis).
(TIF)

S5 Fig Height of participants stratified by diagnosis. In both cohorts of Johannesburg and London, there was no statistically significant difference in height among cases with no-neuropathy (CHANT-ve), neuropathy (CHANT +ve), and neuropathic pain (CHANT+ve, BFP+ve, DN4-I+ve) (Kruskal-Wallis followed by Dunn’s post-hoc analysis).
(TIF)

S6 Fig Waist:Hip ratio of participants stratified by gender and diagnosis. Among females, CHANT+ve neuropathy and neuropathic pain (NeP) (CHANT+ve, BFP+ve, DN4-I+ve) cases had no statistically significant difference in W:H ratio compared to CHANT-ve. Among males, there was no statistically significant difference in W:H ratio between CHANT+ve neuropathy, NeP cases, and CHANT-ve, (Kruskal-Wallis inter-median difference, post-test Dunn).
(TIF)

S7 Fig Female:Male (F:M) ratio stratified by diagnosis. F:M ratio was up to 13 times higher in Johannesburg compared to London. F:M ratio was comparable among those with no neuropathy (CHANT-ve), neuropathy (CHANT+ve), and neuropathic pain (CHANT+ve, BFP+ve, DN4-I+ve).
(TIF)

S8 Fig NRS (Numerical Rating Scale), DAPOS (Depression, Anxiety, and Positive Outlook Scale), BPI (Brief Pain Inventory), and ISI (Insomnia Severity Index) scores in London cohort. NRS, DAPOS, BPI, and ISI showed observable progressively increasing trend from no-neuropathy (CHANT-ve), neuropathy (CHANT+ve), neuropathy with bilateral feet pain (CHANT+ve, BFP+ve), and neuropathic pain (CHANT+ve, BFP+ve, DN4-I+ve). Inter-median BPI (Brief Pain Inventory) difference was statistically significant (*p* < 0.05, Kruskal-Wallis followed by Dunn’s post-hoc analysis) between no-neuropathy CHANT-ve cases and neuropathy CHANT+ve cases; among neuropathy CHANT+ve cases, there was no statistically significant inter-median BPI difference (Kruskal-Wallis followed by Dunn’s post-hoc analysis) between (CHANT+ve, BFP) and (CHANT+ve, BFP, DN4-I+ve).
(TIF)

S9 Fig Frequency of Interpreter Requirement among the study patients in Phase-III (Johannesburg). More than half of the patients required interpreters for the items of ‘*tingling*’, ‘*numbness*’, and ‘*pins and needles*’; this reminds the importance of devising tools that have better comprehensibility for their enhanced utility in field settings where English is a second- or third-language.
(TIF)

S1 Panel Case definition for HIV-SN used by BPNS, UENS, and TCSS. (PDF)

S2 Panel Instruction on how to perform CHANT. (PDF)

S3 Panel Standards Checklist for the CHANT Prototype (COSMIN). (PDF)

S1 Table London CHANT validation study: Inter-median comparison of age, height, and other sociodemographic variables. (PDF)

S2 Table Comparison of DN4-I (Interview part of Douleur Neuropathique 4 Questions) scores among all, no neuropathy (CHANT-), neuropathy (CHANT+), neuropathy with bilateral feet pain (CHANT+, BFP+), and neuropathic pain (CHANT+, BFP+, DN4-I+) patients in London cohort Phase II CHANT internal validation study. There was statistically significant difference between those with neuropathic pain and no neuropathic pain, between neuropathy and no neuropathy, between neuropathy and all, and between neuropathic pain and all (Dunn’s multiple comparison test, *p*< 0.05).
(PDF)

S3 Table London CHANT validation study. Outcomes of NPSI to describe the 9 (CHANT+ve, BFP) patients who showed varied characteristics and severity on all 12 questions. Permanent spontaneous feet pain (question #4 of NPSI) was present among 3 of the 9 (33.33%), while 6/9 (66.67%) stated to have 6–10 episodes of feet pain attacks during the previous 24 hours (question #7).
(PDF)
