# Supplementary figures and images for: Development, Validation, and Field-Testing of an Instrument for Clinical Assessment of HIV-Associated Neuropathy and Neuropathic Pain in Resource-Restricted and Large Population Study Settings
Source: PLoS One. 2016 Oct 20;11(10):e0164994. doi: 10.1371/journal.pone.0164994 (PMC5072607; doi:10.1371/journal.pone.0164994)

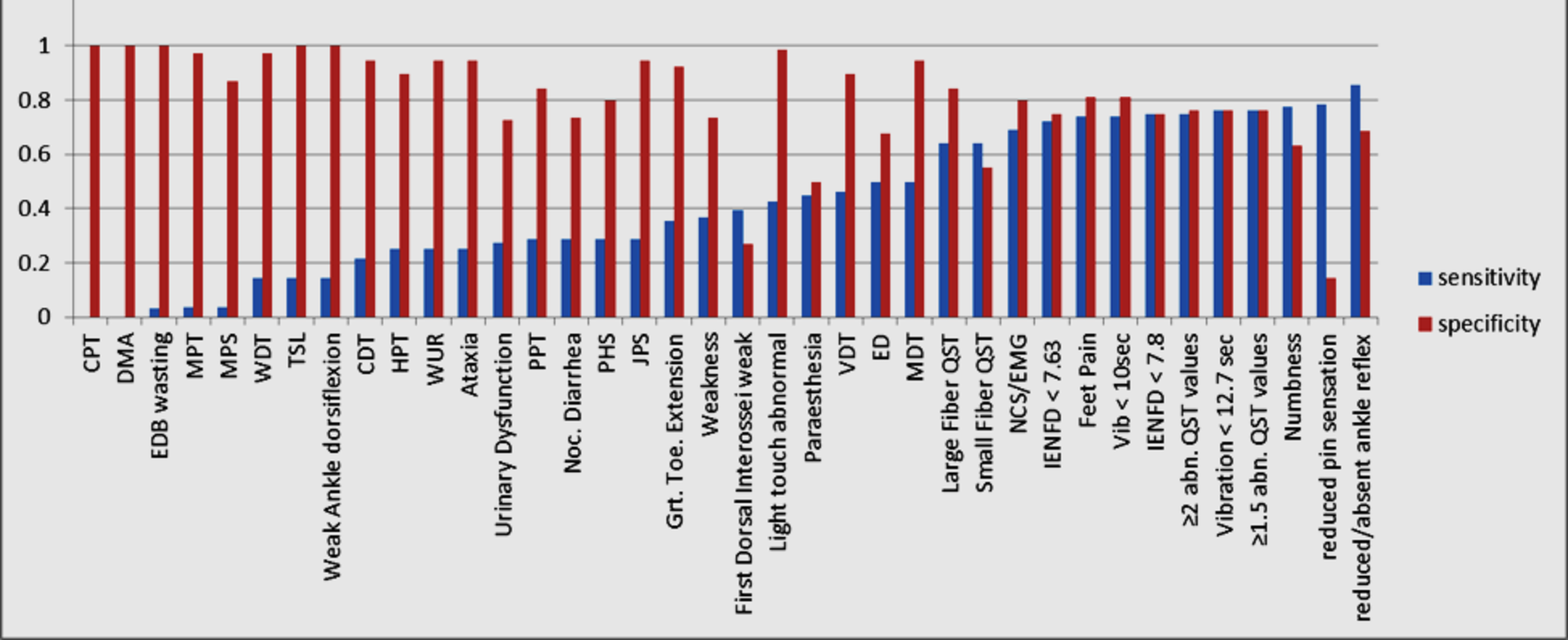

Supplement: S1 Fig — The triumvirate HIV-PINS diagnostic criteria of clinical findings, QST (Quantitative Sensory Testing) abnormalities, and IENFD (Intraepidermal Nerve Fiber Density) results were used as gold-standard. Clinical items with high sensitivity and specificity i.e. feet numbness, feet pain, reduction in ankle reflex and great toe vibration were selected to construct CHANT (Clinical HIV-associated Neuropathy Tool). As shown herewith, these four clinical items performed accurately compared to most of the demanding neuropathy investigations. All items represent measures from bilateral feet. (TIF) [file pone.0164994.s001.tif]

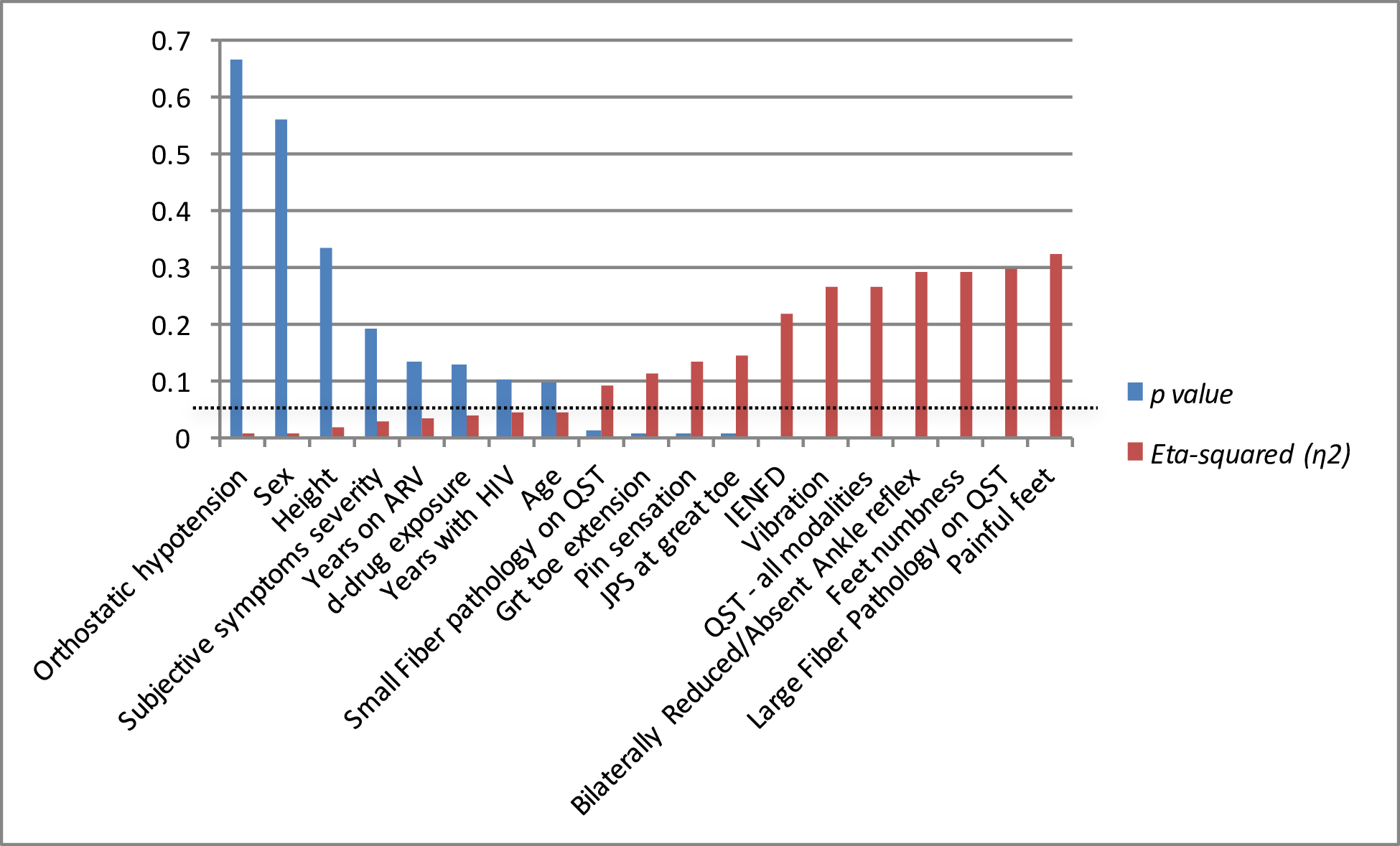

Supplement: S2 Fig — The four clinical items with high sensitivity and specificity i.e. feet numbness, feet pain, reduction in ankle reflex and great toe vibration that were selected to construct CHANT (Clinical HIV-associated Neuropathy Tool) also carried higher accountability effect size compared to most of the demanding neuropathy investigations. All items represent measures from bilateral feet. (TIF) [file pone.0164994.s002.tif]

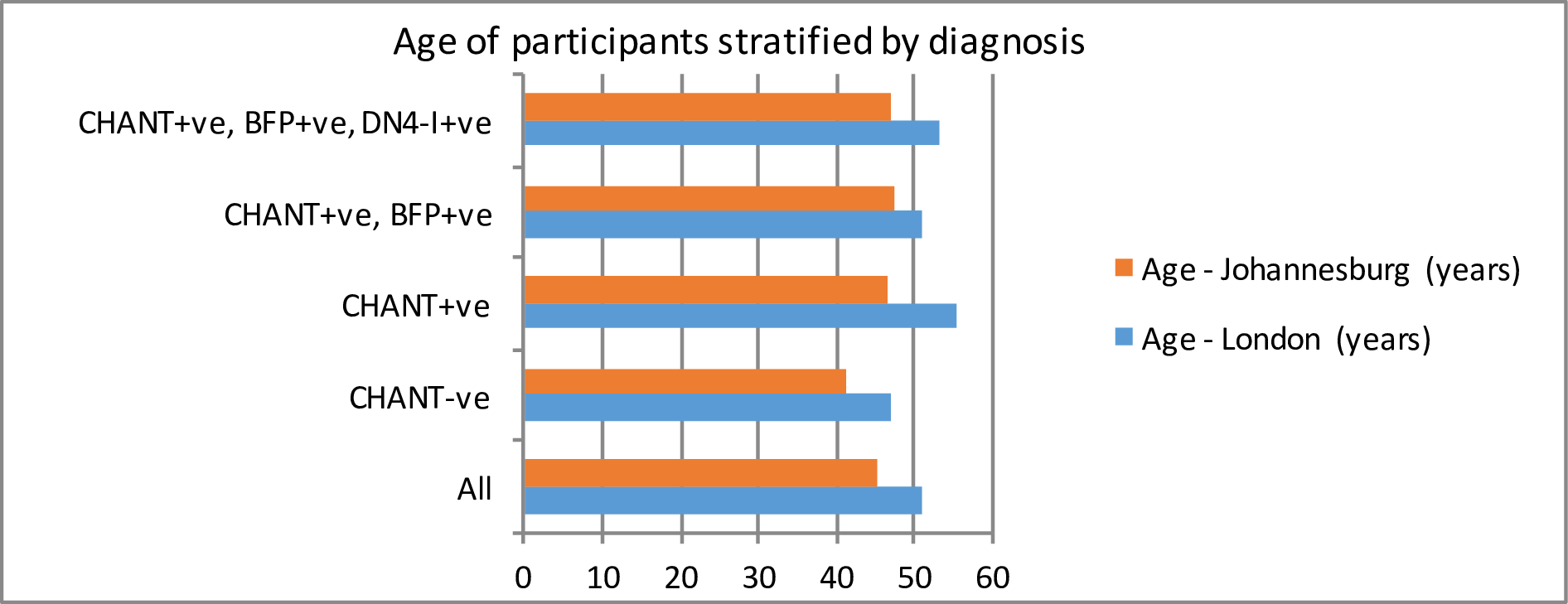

Supplement: S3 Fig — In both cohorts of Johannesburg and London, there was no statistically significant increase in age (years) from no-neuropathy (CHANT-ve), to neuropathy (CHANT +ve), and to neuropathic pain (CHANT+ve, BFP+ve, DN4-I+ve) (Kruskal-Wallis followed by Dunn’s post-hoc analysis). (TIF) [file pone.0164994.s003.tif]

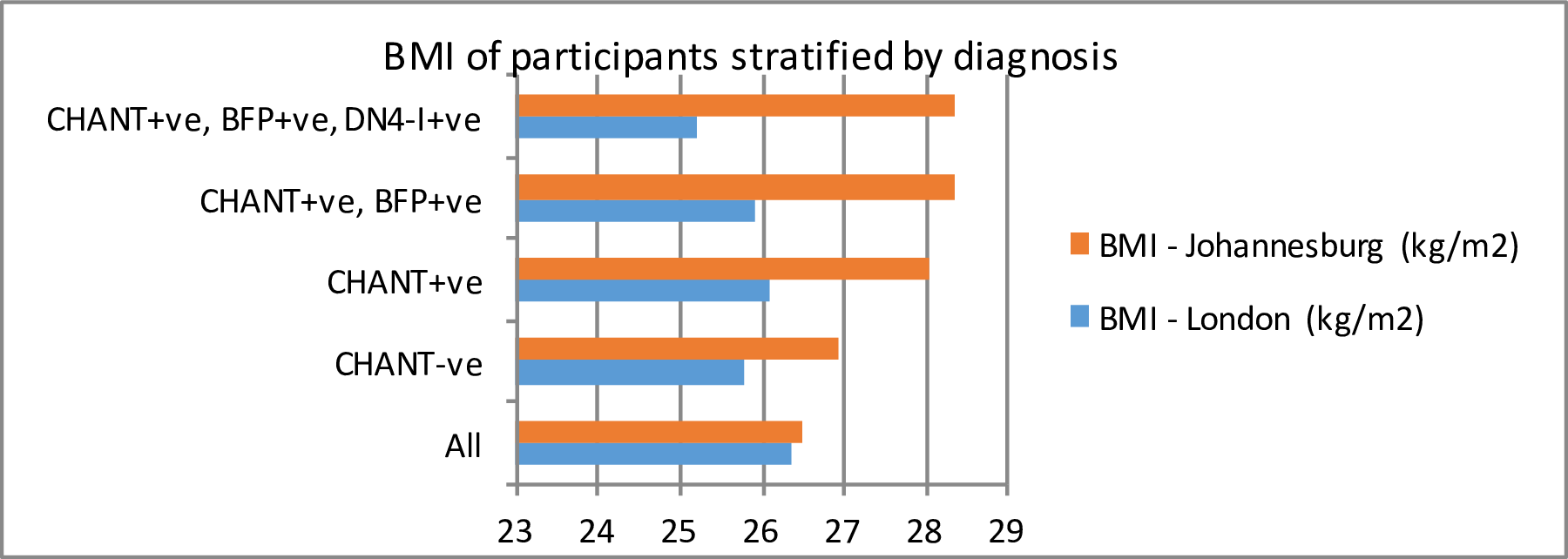

Supplement: S4 Fig — In both cohorts of Johannesburg and London, there was no statistically significant difference in BMI among cases with no-neuropathy (CHANT-ve), neuropathy (CHANT +ve), and neuropathic pain (CHANT+ve, BFP+ve, DN4-I+ve) (Kruskal-Wallis followed by Dunn’s post-hoc analysis). (TIF) [file pone.0164994.s004.tif]

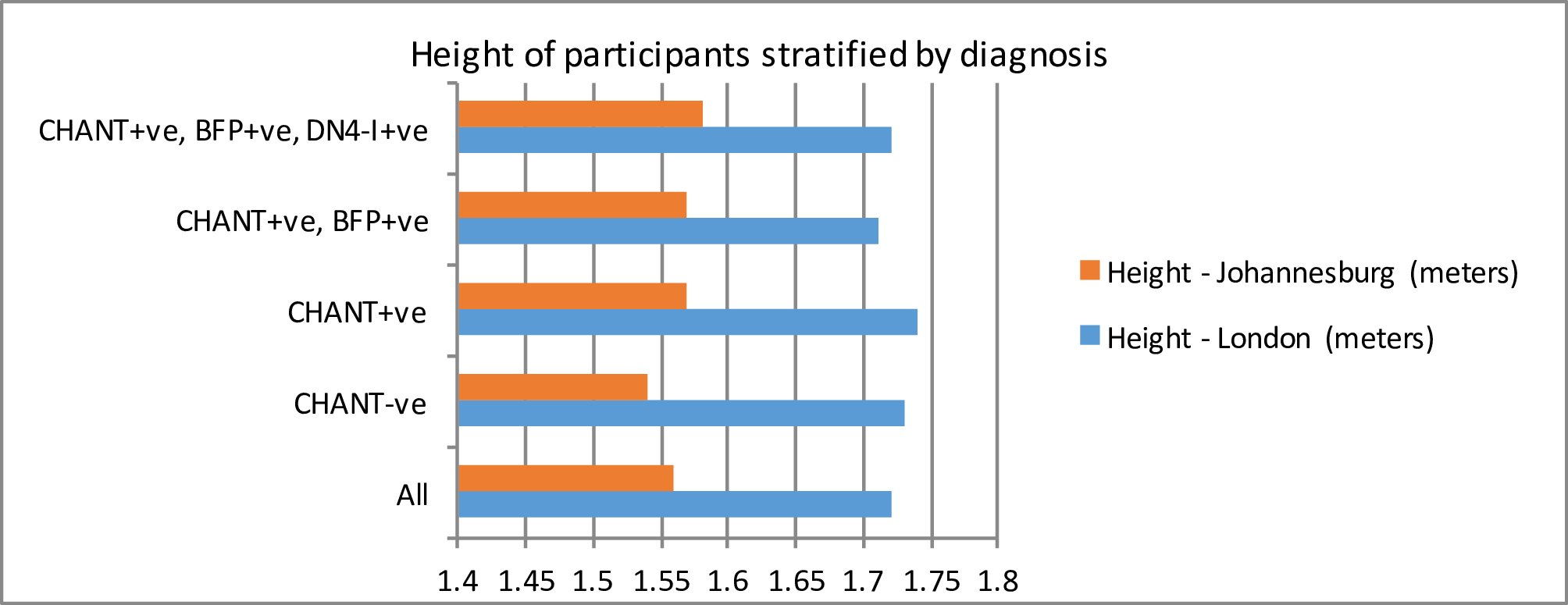

Supplement: S5 Fig — In both cohorts of Johannesburg and London, there was no statistically significant difference in height among cases with no-neuropathy (CHANT-ve), neuropathy (CHANT +ve), and neuropathic pain (CHANT+ve, BFP+ve, DN4-I+ve) (Kruskal-Wallis followed by Dunn’s post-hoc analysis). (TIF) [file pone.0164994.s005.tif]

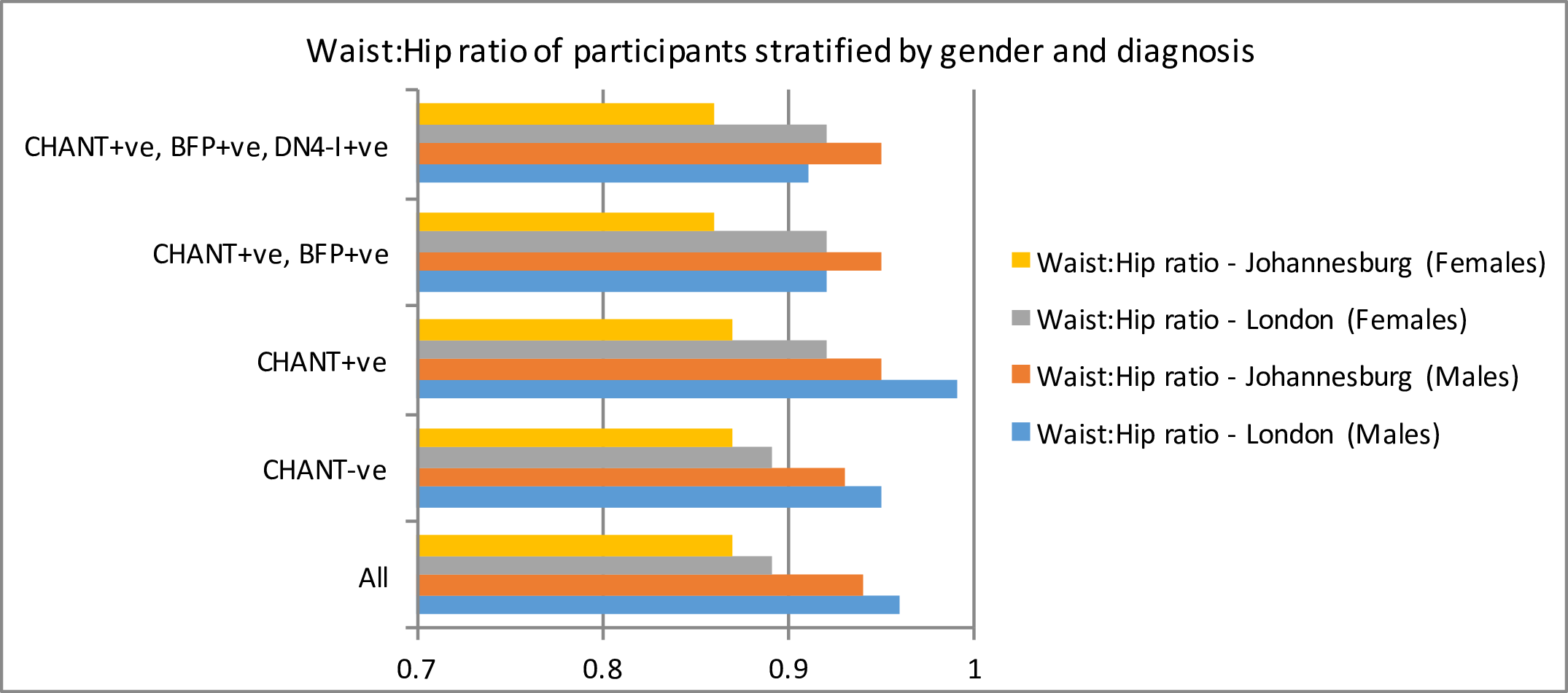

Supplement: S6 Fig — Among females, CHANT+ve neuropathy and neuropathic pain (NeP) (CHANT+ve, BFP+ve, DN4-I+ve) cases had no statistically significant difference in W:H ratio compared to CHANT-ve. Among males, there was no statistically significant difference in W:H ratio between CHANT+ve neuropathy, NeP cases, and CHANT-ve, (Kruskal-Wallis inter-median difference, post-test Dunn). (TIF) [file pone.0164994.s006.tif]

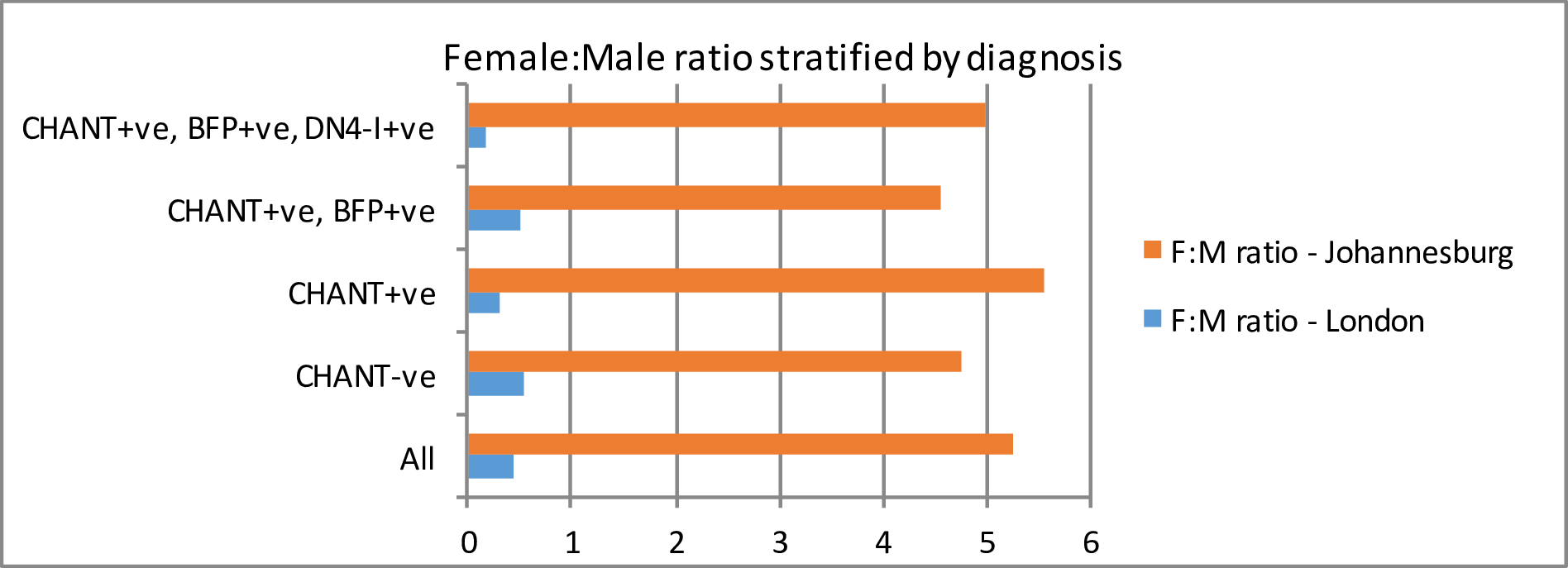

Supplement: S7 Fig — F:M ratio was up to 13 times higher in Johannesburg compared to London. F:M ratio was comparable among those with no neuropathy (CHANT-ve), neuropathy (CHANT+ve), and neuropathic pain (CHANT+ve, BFP+ve, DN4-I+ve). (TIF) [file pone.0164994.s007.tif]

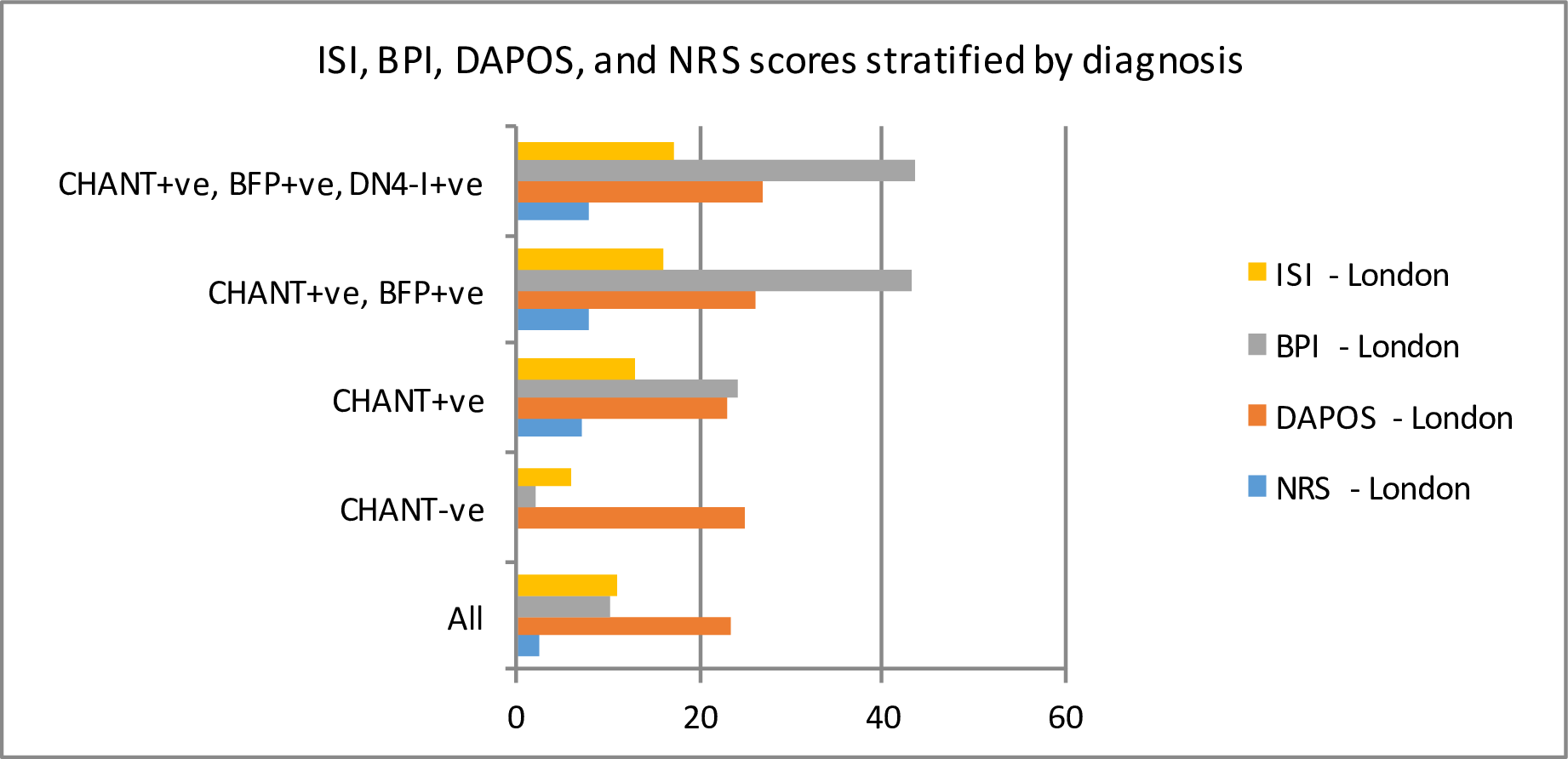

Supplement: S8 Fig — NRS, DAPOS, BPI, and ISI showed observable progressively increasing trend from no-neuropathy (CHANT-ve), neuropathy (CHANT+ve), neuropathy with bilateral feet pain (CHANT+ve, BFP+ve), and neuropathic pain (CHANT+ve, BFP+ve, DN4-I+ve). Inter-median BPI (Brief Pain Inventory) difference was statistically significant (p < 0.05, Kruskal-Wallis followed by Dunn’s post-hoc analysis) between no-neuropathy CHANT-ve cases and neuropathy CHANT+ve cases; among neuropathy CHANT+ve cases, there was no statistically significant inter-median BPI difference (Kruskal-Wallis followed by Dunn’s post-hoc analysis) between (CHANT+ve, BFP) and (CHANT+ve, BFP, DN4-I+ve). (TIF) [file pone.0164994.s008.tif]

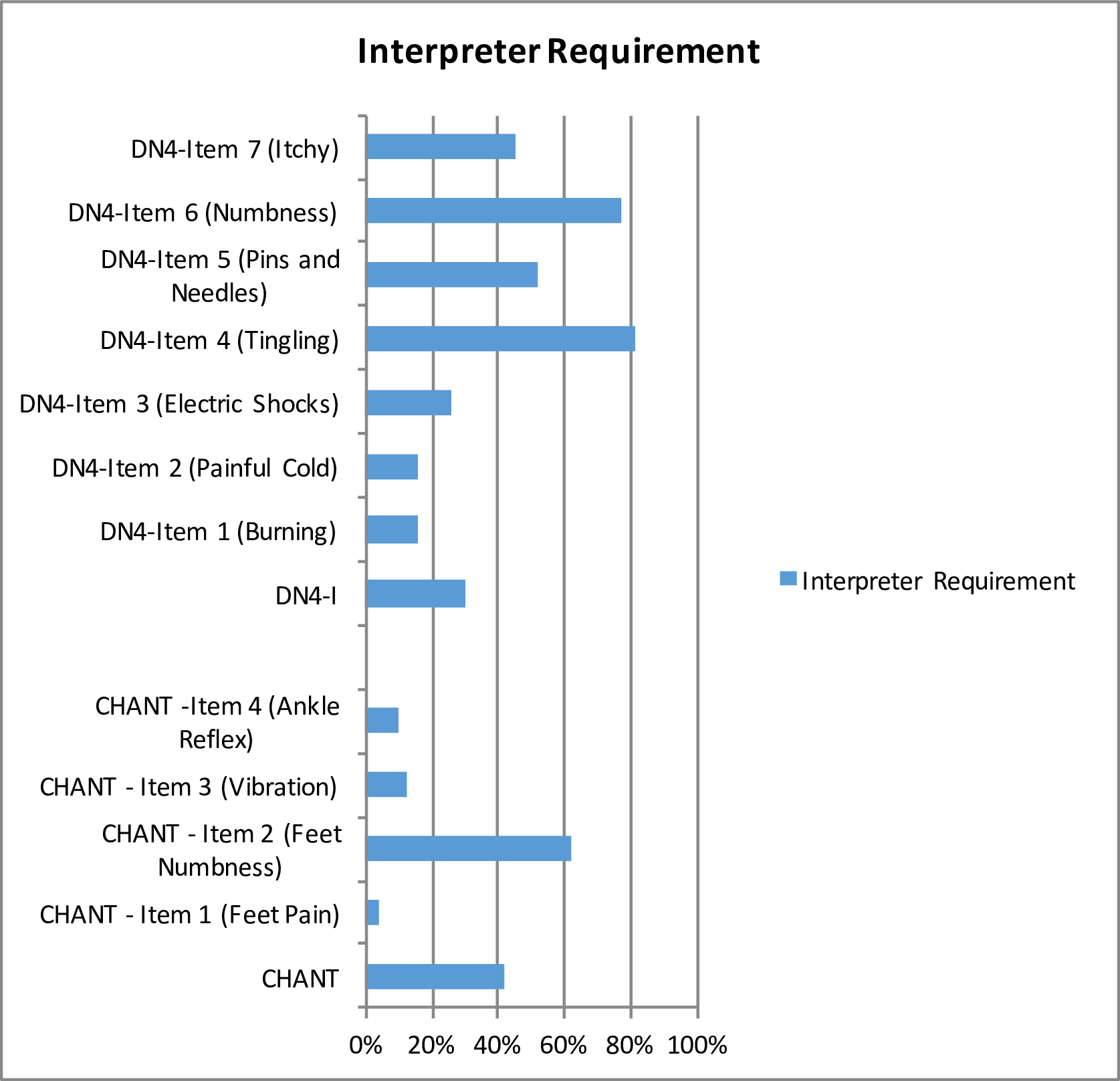

Supplement: S9 Fig — More than half of the patients required interpreters for the items of ‘tingling’, ‘numbness’, and ‘pins and needles’; this reminds the importance of devising tools that have better comprehensibility for their enhanced utility in field settings where English is a second- or third-language. (TIF) [file pone.0164994.s009.tif]
