# Supplementary material for: Development, Validation, and Field-Testing of an Instrument for Clinical Assessment of HIV-Associated Neuropathy and Neuropathic Pain in Resource-Restricted and Large Population Study Settings
Source: PLoS One. 2016 Oct 20;11(10):e0164994. doi: 10.1371/journal.pone.0164994 (PMC5072607; doi:10.1371/journal.pone.0164994)
Supplement: S1 Panel — (PDF) [file pone.0164994.s010.pdf]

S1 Panel: Case definition for HIV-SN used by BPNS, UENS, and TCSS

BPNS: having had at least one of the neuropathic symptoms listed on the BPNS, and additionally were found to have one or more of reduced ankle jerks or reduced vibration sense at the great toe.

UENS: symptom questionnaires, focused physical exam scales, nerve conduction studies (NCS), skin biopsy for intraepidermal nerve fiber density (IENFD) determination, quantitative sudomotor axon reflex testing (QSART), and quantitative sensory testing (QST).

TCSS: sural nerve fiber density
