# Supplementary material for: Development, Validation, and Field-Testing of an Instrument for Clinical Assessment of HIV-Associated Neuropathy and Neuropathic Pain in Resource-Restricted and Large Population Study Settings
Source: PLoS One. 2016 Oct 20;11(10):e0164994. doi: 10.1371/journal.pone.0164994 (PMC5072607; doi:10.1371/journal.pone.0164994)
Supplement: S2 Panel — (PDF) [file pone.0164994.s011.pdf]

S2 Panel: Instruction on how to perform CHANT

Interview part

- **Foot pain:** Ask for the presence of pain over the right foot. Mark *'0'* if absent, *'1'* if present. Repeat similarly for the left foot.
- **Foot numbness:** Ask for the presence of numbness over the right foot. Mark *'0'* if absent, *'1'* if present. Repeat similarly for the left foot.

Neurological Examination part

- **Vibration**: First acquaint the subject with vibration sensation, and then hold the maximally vibrated tuning fork perpendicularly to the dorsum of the great toe at the interphalangeal joint. Extinction of vibration in less than 10 seconds is considered *'reduced'*. To familiarize the subject with the dissipation of vibration, a 128-Hz tuning fork is maximally struck and then held against the clavicle until it is no longer felt, at which point the subject should say *'gone'* to cue the examiner. The number of seconds between the placement of the maximally struck tuning fork on the dorsum of the interphalengeal joint of the great toe and the subject saying *'gone'* are recorded. Write *'1'* for *'reduced'* i.e. extinction of vibration in less than 10 seconds or if absent at all. Write *'0'* for normal i.e. the subject can feel the vibration for $\geq$ 10 seconds. Test both right and left great toes. Make sure the subject is not looking at the test site.
- **Ankle reflex:** With the subject seated, the examiner uses one hand to press upward on the ball of the foot, dorsiflexing the subject's ankle to 90 degrees. Using a reflex hammer, the examiner then strikes the Achilles tendon. Normally, the tendon reflex is felt by the examiner's hand as a plantar flexion of the foot, appearing after a slight delay from the time the Achilles tendon is struck. Use reinforcement by having the subject clench his/her fist before classifying the reflex as *'reduced'* or *'absent'*. Calf muscles contraction without foot plantar flexion is classified as *'reduced'*. Postural changes i.e. kneeling on a chair, prone with knee flexed, supine with hip and knee flexed, need to be applied before classifying as *'reduced'* or *'absent'*. Write *'1'* for *'reduced'* or *'absent'* and *'0'* for *'normal'*.
