# Supplementary material for: Development, Validation, and Field-Testing of an Instrument for Clinical Assessment of HIV-Associated Neuropathy and Neuropathic Pain in Resource-Restricted and Large Population Study Settings
Source: PLoS One. 2016 Oct 20;11(10):e0164994. doi: 10.1371/journal.pone.0164994 (PMC5072607; doi:10.1371/journal.pone.0164994)
Supplement: S3 Panel — (PDF) [file pone.0164994.s012.pdf]

S3 Panel. Standards Checklist for the CHANT Prototype (COSMIN)[1].

- ✓ Measurable: quantifiable scale on all items.
- ✓ Practical: data can be collected on a timely basis and at a reasonable cost, to inform progress and influence decision-making.
- ✓ Reliable: can be measured repeatedly with precision by different investigators.
- ✓ Relevant: helpful in clinical studies of daily practice and neuropathy/neuropathic pain research environment.
- ✓ Management useful: evidence generated is critical to decision-making.
- ✓ Direct: the effect indicators closely track the result it is intended to measure i.e. neuropathy.
- ✓ Sensitive: an early warning of worsening or improving changes in neuropathy.
- ✓ Objective: the neuropathy measure is operationally specific and has no ambiguity.
- ✓ Capable of being disaggregated: data can be broken down by to the different items or some other criteria where appropriate.
- ✓ Capable of being used to require minimal training and expertise to deliver.

1. Mokkink LB, Terwee CB, Patrick DL, Alonso J, Stratford PW, Knol DL, et al. The COSMIN checklist for assessing the methodological quality of studies on measurement properties of health status measurement instruments: an international Delphi study. Quality of life research : an international journal of quality of life aspects of treatment, care and rehabilitation. 2010; **19**(4): 539-49.
