# Supplementary material for: Development, Validation, and Field-Testing of an Instrument for Clinical Assessment of HIV-Associated Neuropathy and Neuropathic Pain in Resource-Restricted and Large Population Study Settings
Source: PLoS One. 2016 Oct 20;11(10):e0164994. doi: 10.1371/journal.pone.0164994 (PMC5072607; doi:10.1371/journal.pone.0164994)
Supplement: S2 Table — There was statistically significant difference between those with neuropathic pain and no neuropathic pain, between neuropathy and no neuropathy, between neuropathy and all, and between neuropathic pain and all (Dunn’s multiple comparison test, p< 0.05). (PDF) [file pone.0164994.s014.pdf]

**Compared cohorts**	**Dunn's Multiple Comparison Test *p*-value**
DN4-I (CHANT+, BFP+, DN4-I+) vs DN4-I (CHANT+, BFP+)	$p > 0.05$
DN4-I (CHANT+, BFP+, DN4-I+) vs DN4-I (CHANT+)	$p > 0.05$
DN4-I (CHANT+, BFP+, DN4-I+) vs DN4-I (CHANT-)	$p \leq 0.001$
DN4-I (CHANT+, BFP+, DN4-I+) vs DN4-I, all	$p \leq 0.05$
DN4-I (CHANT+, BFP+) vs DN4-I (CHANT+)	$p > 0.05$
DN4-I (CHANT+, BFP+) vs DN4-I (CHANT-)	$p \leq 0.001$
DN4-I (CHANT+, BFP+) vs DN4-I, all	$p \leq 0.05$
DN4-I (CHANT+) vs DN4-I (CHANT-)	$p \leq 0.001$
DN4-I (CHANT+) vs DN4-I, all	$p \leq 0.05$
DN4-I (CHANT-) vs DN4-I, all	$p > 0.05$
