# Supplementary material for: Development, Validation, and Field-Testing of an Instrument for Clinical Assessment of HIV-Associated Neuropathy and Neuropathic Pain in Resource-Restricted and Large Population Study Settings
Source: PLoS One. 2016 Oct 20;11(10):e0164994. doi: 10.1371/journal.pone.0164994 (PMC5072607; doi:10.1371/journal.pone.0164994)
Supplement: S3 Table — Outcomes of NPSI to describe the 9 (CHANT+ve, BFP) patients who showed varied characteristics and severity on all 12 questions. Permanent spontaneous feet pain (question #4 of NPSI) was present among 3 of the 9 (33.33%), while 6/9 (66.67%) stated to have 6–10 episodes of feet pain attacks during the previous 24 hours (question #7). (PDF) [file pone.0164994.s015.pdf]

NPSI among CHANT+ve AND Body Map +ve	0	1	2	3	4	5	6	7	8	9	10
Q1: Does your pain feel like burning?	✓✓✓✓		✓	✓		✓		✓	✓		
Q2: Does your pain feel like squeezing?	✓✓✓✓<br>✓		✓			✓				✓	
Q3: Does your pain feel like pressure?	✓✓	✓	✓	✓				✓	✓		✓
Q5: Does your pain feel like electric shocks?	✓✓✓			✓	✓	✓		✓<br>✓	✓		✓
Q6: Does your pain feel like stabbing?	✓✓✓		✓	✓			✓		✓	✓<br>✓	
Q8. Is your pain provoked or increased by brushing on the painful area?	✓✓✓✓		✓✓						✓	✓	✓
Q9. Is your pain provoked or increased by pressure on the painful area?	✓✓			✓			✓	✓	✓<br>✓		✓<br>✓
Q10. Is your pain provoked or increased by contact with something cold on the painful area?	✓✓✓✓		✓✓			✓			✓<br>✓		
Q11. Do you feel pins and needles?	✓	✓		✓		✓<br>✓	✓<br>✓<br>✓		✓		✓
Q12. Do you feel tingling?	✓	✓✓			✓		✓	✓<br>✓	✓		✓
	Between 1 - 3h	Between 4 and 7h	Between 8 and 12h	Permanently							
Q4: *During the past 24 hours*, your spontaneous pain has been present:	✓✓✓	✓✓	✓	✓✓✓							
	Between 1 and 5	Between 6 and 10	Between 11 and 20	More than 20							
Q7: *During the past 24 hours*, how many of these pain attacks have you had?	✓✓	✓✓✓✓<br>✓✓		✓							
